# A Cue-Level Fusion Framework for Robust Soccer Player Tracking: Integrating Appearance, Motion, and Geometric Cues for Adaptive Tracklet Linking

**DOI:** 10.3390/jimaging12100512

**Published:** 2026-10-14

**Authors:** Qingchun Hu, Tingxi Li, Xintao Han, Yufei Tang

**Affiliations:** Department of Computer Science, East China University of Science and Technology, Shanghai 200237, China

**Keywords:** cue-level fusion, motion-appearance gating, multi-object tracking, soccer video analysis, tracklet linking, adaptive gating, multi-cue veto cascade, re-identification

## Abstract

Cue-level fusion of complementary information channels—appearance for identity, motion for trajectory, and geometry for spatial consistency—is central to multi-object tracking in soccer broadcast videos, yet existing tracking-by-detection frameworks are validated mainly on pedestrian data. This paper proposes a cue-level fusion framework for soccer player tracking on the SoccerNet SN-Tracking dataset (57 train, 49 test sequences). Fusion is performed at two levels: (i) motion-appearance gating during online association, fusing Mahalanobis distance and cosine distance through gated cascade matching; and (ii) cue-level post-processing, jointly fusing temporal, spatial, and geometric cues for tracklet linking. Our contributions are: (1) a systematic cue-contribution analysis over ten configurations, quantifying independent and interaction effects—including the pure contribution of the OSNet appearance model—and showing that appearance is the dominant contributor (+19.00 HOTA) while the original linking cue suffers from gain saturation on high-quality trackers; (2) a hyperparameter sensitivity analysis identifying soccer-specific best-performing configurations, including motion retention at 25 frames (~1 s at 25 fps), which is fixed by a pre-specified detection-gap rule established on the training split and is significantly better than neighboring values on the test split (20 → 25: +2.80, t(48) = 5.57, *p* < 0.001, Cohen’s d = +0.80; 25 → 30: −3.12, *p* < 0.001); and (3) a multi-cue veto cascade (MCVC), a training-free and strictly subtractive team-color equivalence-class veto that overcomes saturation by rejecting cross-team links—including those involving outlier entities such as referees and the ball—instead of re-scoring them, achieving gains of +2.22 HOTA and +4.04 AssA over the original AFLink (paired per-sequence test against the no-veto control, *p* < 0.001 on both splits). The best configuration achieves 64.31% HOTA on the test set, providing a configurable cue-level baseline for soccer video tracking.

## 1. Introduction

Soccer, as the world’s most popular sport, generates massive volumes of broadcast video data with significant tactical analysis and commercial value. Automatically extracting player trajectories and tactical information from long, unstructured raw video has become an active research area in computer vision. Multi-object tracking (MOT), the core technology underpinning such analysis, aims to continuously localize multiple players across video sequences while maintaining identity consistency.

MOT in soccer broadcast videos faces a unique set of challenges that differentiate it from the well-studied pedestrian tracking domain [1,2,3,4]. First, broadcast cameras undergo extensive panning, zooming, and rotation to follow play, causing severe inter-frame background displacement that invalidates simple motion models. Second, players exhibit fast, non-linear motion patterns—sprints, sudden direction changes, and aerial challenges—that violate the constant-velocity assumption of standard Kalman filters. Third, teammates wearing highly similar jerseys create severe appearance ambiguity, making identity preservation extremely difficult during dense interactions. Fourth, frequent occlusions—from other players, the referee, or camera perspective changes—cause detection dropouts that fragment trajectories. Finally, significant scale variation (distant players may occupy only a few pixels) poses challenges for both detection and association.

From a cue-level perspective, soccer MOT inherently involves three distinct information cues that must be effectively fused: (i) the *visual appearance* cue—deep Re-ID features that discriminate between players sharing similar team jerseys; (ii) the *motion dynamics* cue—Kalman filter predictions and camera motion compensation that model trajectory evolution; and (iii) the *geometric structure* cue—bounding box shape, area, and aspect ratio that encode spatial consistency. Existing tracking-by-detection frameworks such as SORT [5], DeepSORT [6], BoT-SORT [7], and StrongSORT [8] incorporate elements of these cues, yet these frameworks were designed and validated primarily on pedestrian data. The systematic analysis of how these cues individually and jointly contribute to tracking quality—particularly in the demanding sports domain—remains largely unanswered.

Three critical questions arise for cue-level soccer tracking:

1. **Cue contribution:** How do the appearance, motion, and geometric cues individually and jointly contribute to tracking quality in soccer videos? Existing studies report module performance on pedestrian data, but the unique characteristics of soccer (similar appearances, dynamic camera) may fundamentally alter these cue contributions.
2. **Fusion configuration:** Are the fusion hyperparameters optimized for pedestrian tracking (e.g., motion model retention max_age = 30 at 30 fps) appropriate for soccer video at 25 fps? Systematic sensitivity analysis is needed to identify soccer-specific best-performing fusion settings.
3. **Post-processing gain saturation:** AFLink [8] was designed to improve tracking by globally linking fragmented trajectories through spatio-temporal fusion. However, when the base tracker already produces high-quality outputs, AFLink’s gains diminish—a phenomenon we term *gain saturation*. How can cue-level fusion in post-processing be made adaptive to overcome this saturation?

To address these questions, this paper proposes a systematic cue-level fusion analysis and an adaptive tracklet linking mechanism for soccer video tracking on the SoccerNet SN-Tracking dataset [9]. Our framework performs fusion at two levels: motion-appearance gating during online association (fusing motion and appearance cues) and cue-level post-processing (fusing temporal, spatial, and geometric cues for tracklet linking). Our main contributions are as follows:

- **Systematic cue contribution analysis:** We design ten configurations (A–J) that progressively integrate the motion (ECC), appearance (Re-ID: ResNet50 vs. OSNet), and post-processing (AFLink) cues, quantifying each component’s independent and interaction effects. Our analysis reveals that the appearance cue is the dominant contributor (+19.00 HOTA), while the linking cue produces zero gain on ResNet50-based trackers and modest gains with OSNet—a finding that motivates our cue-level adaptive improvement and, in a second step, the veto cascade that defines the final configuration (J).
- **Soccer-specific fusion optimization:** We conduct sensitivity analysis on 13 parameters on the full 57-sequence train set and the full 49-sequence test set. Key findings include the identification of a best-performing motion retention at 25 frames (~1 s), the superiority of lower detection confidence thresholds (conf = 0.25) for sports scenarios, the insignificance of spatial distance and feature queue size within reasonable ranges, and the dominance of trajectory reliability over shape continuity in the gate signal.
- **Quality-aware cue-level tracklet linking with a multi-cue veto cascade:** We propose a *training-free*, *strictly subtractive* multi-cue veto cascade (MCVC) that rejects geometrically plausible but semantically impossible cross-team links using team-color equivalence classes, including outlier entities such as referees and the ball. MCVC is combined with an improved AFLink whose adaptive gating and two-factor scoring mechanism establishes the admissible search window; a component-wise ablation shows that widening that window alone accounts for the bulk of the recovery, while the adaptive gate is not statistically significant and is retired in the final configuration. The final configuration achieves statistically significant gains over the original AFLink (HOTA +2.22%, AssA +4.04%, *p* < 0.001 against the no-veto control on both splits), and the cascade attains 79.8% of the oracle upper bound on the test split.

Before detailing our approach, it is important to clarify what we mean by “fusion” in our tracking context. Unlike heterogeneous-sensor fusion that combines physically distinct inputs (e.g., RGB images, depth maps, audio), our framework treats appearance, motion, and geometric shape as three distinct *information cues* derived from the same visual input—each encoding a complementary aspect of player identity and dynamics. This conceptualization follows the broader usage of “cue” in tracking the literature, where a cue is any measurable signal that contributes to identity or trajectory estimation; appearance features capture visual identity, motion dynamics encode temporal evolution, and geometric cues describe spatial structure. By systematically combining these three cues through both *online motion-appearance gating* (cascade matching during association) and *offline cue-level post-processing* (joint temporal-spatial-geometric scoring for tracklet linking), we obtain a principled multi-cue system whose synergy differs from single-cue or loosely coupled trackers.

The remainder of this paper is organized as follows. Section 2 reviews related work in MOT, sports tracking, cue-level fusion, and post-processing. Section 3 describes our cue-level fusion methodology, including the framework architecture, the adaptive AFLink, and the multi-cue veto cascade. Section 4 details the experimental setup. Section 5 presents results and discussion. Section 6 concludes with future directions.

## 2. Related Work

### 2.1. Multi-Object Tracking

The tracking-by-detection paradigm dominates modern MOT. SORT [5] established a simple baseline using a Kalman filter for motion prediction and Intersection-over-Union (IoU) for data association via the Hungarian algorithm. DeepSORT [6] extended SORT by incorporating a deep appearance descriptor (a 128-dimensional CNN feature vector) and fusing motion (Mahalanobis distance) with appearance (cosine distance) costs through a weighted gate, reducing identity switches by approximately 45% on MOT16.

BoT-SORT [7] advanced the framework with several key improvements: (1) camera motion compensation via Enhanced Correlation Coefficient (ECC) maximization, which estimates inter-frame transformation matrices to correct Kalman filter predictions; (2) a refined Kalman state vector directly estimating bounding box width and height (rather than aspect ratio) for better shape fitting; and (3) a min-based motion-appearance fusion strategy. StrongSORT [8] took a different approach, systematically integrating six improvements (BoT, ECC, NSA, EMA, MC, woC) into the DeepSORT framework and proposing two plug-and-play post-processing algorithms: AFLink (Appearance-Free Link) for global tracklet association and GSI (Gaussian-smoothed Interpolation) for trajectory smoothing.

Despite these advances, BoT-SORT and StrongSORT were validated primarily on pedestrian datasets. Their component-level behavior in sports scenarios—characterized by rapid camera motion and appearance similarity—remains underexplored. Recent methods such as MeMoSORT [10] have introduced memory-assisted filtering and motion-adaptive metrics, but again in the pedestrian context.

### 2.2. Multi-Object Tracking in Sports

Sports video tracking presents distinct challenges that have motivated domain-specific solutions. DCTracker [11] addresses dual-view tracking in soccer events through cascade association, handling the perspective changes common in broadcast production. Deep HM-SORT [12] enhances sports tracking by integrating deep features, harmonic mean IoU, and Expansion IoU to better handle fast-moving athletes. SportMamba [13] employs state-space models for adaptive non-linear motion modeling, explicitly targeting the non-constant velocity patterns in team sports. SportsMOT [14] is a large-scale multi-sport MOT dataset covering basketball, volleyball, and football, and it explicitly characterizes sports MOT by fast and variable-speed motion and similar yet distinguishable appearance.

The SoccerNet benchmark [1,2] has become the standard evaluation platform for soccer video analysis, with its Tracking Challenge attracting numerous participating teams. The MOT4MOT team [15] achieved commendable results (3rd place in the 2023 challenge) using a YOLOv8l + DeepOC-SORT scheme. More recently, Golovkin et al. [16] achieved state-of-the-art game state reconstruction by combining advanced detection and tracking with pitch geometric constraints. In action understanding, the SoccerNet Ball Action Anticipation task [17] has introduced new metrics (mAP@delta, mAP@infty) and the FAANTRA baseline, broadening the scope of soccer video analysis beyond tracking.

However, existing sports MOT studies typically report end-to-end results without isolating component contributions or analyzing hyperparameter sensitivity, making it difficult for practitioners to identify which modules matter most and how to configure them. Among the various tracking components, post-processing techniques such as tracklet linking play a critical yet under-analyzed role in sports scenarios—we next review this line of work.

### 2.3. Tracklet Linking and Post-Processing

Post-processing aims to repair trajectory interruptions caused by detection failures and association errors. StrongSORT’s AFLink [8] performs global tracklet association using only spatio-temporal information, without relying on appearance features. Its architecture consists of three stages: (1) a rule-based coarse filtering stage using fixed thresholds on temporal gap and spatial distance, (2) a neural network scoring stage for candidate pairs, and (3) Hungarian matching for best-performing assignment. AFLink yields greater gains for weaker trackers but provides diminishing returns as base tracker quality improves.

GSI [8] complements AFLink by applying Gaussian process regression to smooth trajectories and interpolate missing detections, improving temporal consistency. Alternative post-processing approaches include global tracklet association methods [18] that use graph-based optimization, and interpolation techniques based on motion models. More recent end-to-end trackers such as MOTRv2 [19] bootstrap multi-object tracking from a pretrained object detector and learn association end-to-end, but require large labeled datasets and are currently less common in sports MOT than tracking-by-detection frameworks; we therefore do not adopt them in this work. Real-time soccer-specific deployments have also been reported on commodity hardware [20].

A key limitation of the original AFLink is its use of fixed thresholds (temporal gap ≤ 30, spatial distance ≤ 75), which are suboptimal across different tracker quality levels and scene characteristics. When the base tracker is high-quality (producing few, long, reliable tracklets), these conservative thresholds prevent any candidate pairs from passing coarse filtering, resulting in zero gain—a phenomenon we analyze in detail in Section 5.1. Our work addresses this limitation through adaptive gating.

### 2.4. Cue-Level Fusion in Visual Tracking

Cue-level fusion aims to combine complementary information channels that, taken individually, are insufficient for a robust prediction. Baltršaitis et al. [21] provided a comprehensive taxonomy of multimodal machine learning, categorizing fusion into early (feature-level), late (decision-level), and hybrid approaches. In visual tracking, cue fusion naturally arises because robust identity preservation requires combining cues that are individually insufficient: motion models predict where an object will be, appearance models describe what it looks like, and geometric models encode how it is shaped. We use the term “cue” rather than “modality” because all three signals (appearance, motion, geometry) are derived from the same visual input; in the strict multimodal-learning sense, they would belong to a single modality (RGB video).

DeepSORT [6] pioneered motion-appearance fusion in MOT by combining Mahalanobis distance (motion) with cosine distance (appearance) through a gated weighting scheme. BoT-SORT [7] refined this with a min-based fusion that selects the more reliable cue per association pair, and added camera motion compensation as an auxiliary motion cue. StrongSORT [8] further integrated multiple cues through its six-component framework (BoT, ECC, NSA, EMA, MC, woC), where EMA (Exponential Moving Average) updates the appearance state of a track over time and NSA (Noise Scale Adaptive Kalman filter) rescales the measurement-noise covariance of the motion branch by the detection confidence, so that low-confidence detections are trusted less in the state update.

Beyond binary motion-appearance fusion, recent works have explored richer cue integration. In particular, deep association metrics learn a shared embedding space across visual cues (motion vs. appearance) as an alternative to heuristic gating; representative pedestrian-domain examples include the original DeepSORT association head [6] and the appearance-free BoT-SORT refinement [7]. We treat such learned-embedding approaches as conceptually adjacent to, but distinct from, the cue-level scoring we use, which combines hand-crafted temporal, spatial, and geometric cues with a strictly subtractive veto layer. In sports analytics, multi-source analyses have incorporated additional inputs such as player pose [22], ball trajectory [17], and pitch geometry [16], though these typically augment downstream analysis rather than core tracking. GTA [18] performs global tracklet association using graph-based optimization that fuses temporal, spatial, and appearance cues—a direction conceptually related to our adaptive AFLink, though optimized for different scenarios.

Despite these advances, three aspects of cue-level fusion in sports MOT remain underexplored: (1) quantitative analysis of relative cue contributions in the sports domain, where appearance ambiguity and dynamic cameras may shift cue importance; (2) adaptive fusion that adjusts cue weights based on trajectory quality and scene context; and (3) geometric cue integration in post-processing, which existing AFLink-type methods handle only through fixed, non-adaptive rules. Our work addresses all three gaps.

### 2.5. Research Gaps

Table 1 summarizes major related studies from recent years. Based on this review, we identify three gaps in cue-level fusion for soccer video MOT: (1) lack of quantitative analysis of interactions among the motion (ECC), appearance (Re-ID), and geometric linking (AFLink) cues in soccer scenarios; (2) fusion hyperparameters inherited from pedestrian defaults without systematic validation on soccer data; (3) insufficient discussion of the geometric linking cue’s marginal effects on high-quality trackers and a lack of adaptive cue fusion in post-processing. This paper aims to fill these gaps through systematic experimentation and targeted methodological improvement.

## 3. Methodology

### 3.1. Cue-Level Fusion Architecture Overview

**Figure 1:**
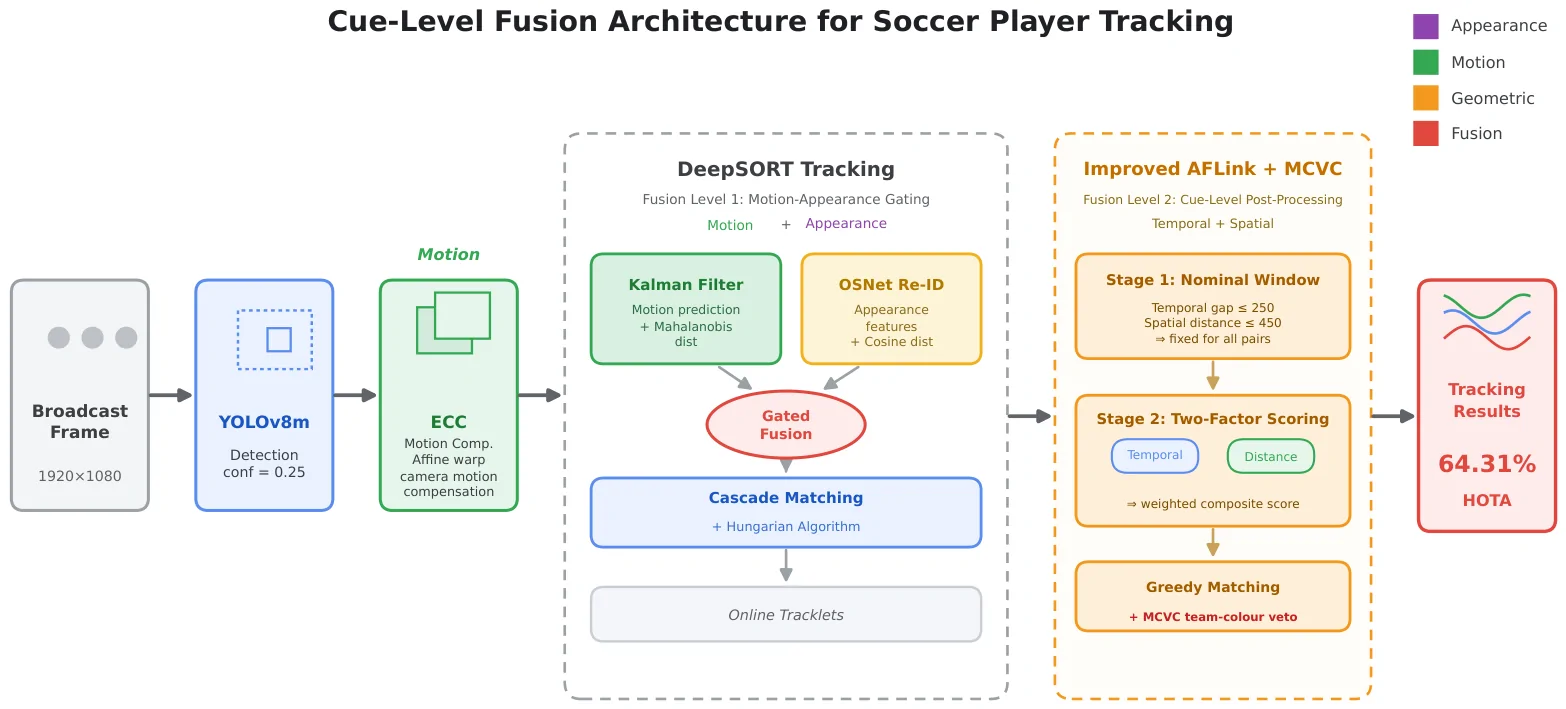
Cue-level fusion architecture overview. The pipeline flows from left to right: YOLOv8m detection → ECC motion compensation (motion cue M) → DeepSORT tracking with motion-appearance gating (motion M × appearance A) → improved AFLink post-processing (two-factor temporal–spatial scoring + MCVC veto) → tracking results. The cues are fused at two levels: motion-appearance gating (Level 1) and cue-level post-processing (Level 2).

Our tracking system follows the tracking-by-detection paradigm and is designed as a cue-level fusion framework, as illustrated in Figure 1. The pipeline consists of four stages that progressively fuse three information cues: (1) object detection using YOLOv8m, providing initial localization; (2) camera motion compensation via ECC, refining the motion cue; (3) online tracking with DeepSORT incorporating Re-ID features, performing motion-appearance gating between the motion and appearance cues through gated cascade matching; and (4) post-processing with the improved AFLink, scoring candidate links with the two-factor temporal–spatial rule and filtering them with the multi-cue veto cascade. Each stage is configurable, enabling systematic cue-contribution analysis.

Formally, the three cues are defined as:

- **Appearance cue *A*:** deep feature vectors $f_{i}\in R^{d}$ extracted by the Re-ID model, encoding visual identity.
- **Motion cue** ***M*****:** Kalman filter state predictions ${\hat{x}}_{t|t-1}$ and ECC-compensated transforms $M_{t}$, encoding temporal dynamics.
- **Geometric cue** ***G*****:** bounding box shape descriptors (A,r) comprising area and aspect ratio, encoding spatial structure.

These cues are fused at two levels: (i) online motion-appearance gating (Section 3.3), where *M* and *A* are combined through gated cascade matching; and (ii) offline cue-level post-processing (Section 3.6), where temporal, spatial, and geometric cues are jointly fused for tracklet linking. The overall architecture is illustrated in Figure 1. We design ten configurations (A–J) that progressively integrate the three tracking cues—motion (ECC), appearance (Re-ID), and geometric linking (AFLink)—as shown in Section 5.1.

### 3.2. Object Detection

We employ YOLOv8m [23] as the detector, which provides a favorable balance between speed and accuracy. The detector outputs bounding boxes and confidence scores for each frame. Two key hyperparameters control detection behavior:

- **Confidence threshold (conf):** Filters detections below this score. We find that soccer scenarios benefit from a lower threshold (0.25) compared to the typical pedestrian setting (0.5–0.6) [23,24], because distant players produce low-confidence detections that are still valid tracking targets.
- **Input resolution (imgsz):** Determines the resolution at which the detector processes each frame. Higher resolution preserves fine details for small-target detection but increases computational cost.

### 3.3. Online Tracking with Motion-Appearance Gating

The DeepSORT tracker maintains a set of Kalman filter-based trackers, each corresponding to an active trajectory. The Kalman filter uses an 8-dimensional state vector:

(1) $$x={\left[u,v,\gamma ,h,\dot{u},\dot{v},\dot{\gamma },\dot{h}\right]}^{T}$$

where (u,v) is the bounding box center, (γ,h) are the aspect ratio and height, and the remaining four components are their respective velocities. This formulation follows DeepSORT [6], which uses the aspect ratio γ and height *h* to parameterize the bounding box.

Data association employs a *motion-appearance gating* strategy via cascade matching [6]: for each cascade level, the motion cue *M* (Mahalanobis distance from Kalman predictions) and the appearance cue *A* (cosine distance from Re-ID features) are fused through a gating mechanism. The motion cost $d^{\left(m\right)}$ and appearance cost $d^{\left(a\right)}$ are combined:

(2) $$d=\left\{\begin{array}{cc} d^{\left(a\right)}, & \mathrm{if}\;d^{\left(m\right)}\leq \tau_{m}\;(\mathrm{motion}\;\mathrm{gate}\;\mathrm{passes})\\ \infty , & \mathrm{otherwise} \end{array}\right.$$

Here $\tau_{m}=9.4877$ is the 95th percentile of the chi-squared distribution with four degrees of freedom, following the DeepSORT default [6]; the same value is reused across all experiments without retuning. This gated fusion uses the motion cue as a *hard filter* to eliminate spatially implausible candidates, then relies on the appearance cue for fine-grained discrimination among the remaining candidates—a design particularly suited for soccer, where teammates’ similar appearances require the motion gate to first prune the candidate space. Key tracking hyperparameters that control this fusion include:

- max_age: Maximum number of frames a trajectory survives without matched detections before deletion.
- n_init: Number of consecutive detections required to confirm a new trajectory, filtering flickering false positives.
- match_thresh: Maximum cost for a valid match in the Hungarian algorithm [25].
- max_dist: Maximum spatial distance for candidate matching.
- max_iou_dist: Maximum IoU distance for second-stage matching.

### 3.4. Camera Motion Compensation

Soccer broadcast cameras undergo extensive panning and zooming, causing significant inter-frame coordinate shifts. We employ ECC (Enhanced Correlation Coefficient) maximization [7,26] for camera motion compensation. ECC estimates an affine transformation between consecutive frames by maximizing the correlation coefficient of aligned image regions. The resulting 2×3 affine matrix is extended to a 3×3 homogeneous matrix ${\tilde{M}}_{t}$:

(3a) $${\tilde{M}}_{t}=\left[\begin{array}{ccc} m_{11} & m_{12} & m_{13}\\ m_{21} & m_{22} & m_{23}\\ 0 & 0 & 1 \end{array}\right]$$

The 8-dimensional Kalman state ${\hat{x}}_{t|t-1}={\left[u,v,\gamma ,h,\dot{u},\dot{v},\dot{\gamma },\dot{h}\right]}^{T}$ is then transformed by an 8×8 block-diagonal matrix $T_{t}$:

(3b) $${\hat{x}}_{t|t-1}^{\prime }=T_{t}\;{\hat{x}}_{t|t-1},\;T_{t}=\left[\begin{array}{cc} A_{t} & 0_{4\times 4}\\ 0_{4\times 4} & I_{4\times 4} \end{array}\right]$$

where $A_{t}$ carries the affine part of ${\tilde{M}}_{t}$ and leaves the scale components unchanged:

(3c) $$A_{t}=\left[\begin{array}{cccc} m_{11} & m_{12} & m_{13} & 0\\ m_{21} & m_{22} & m_{23} & 0\\ 0 & 0 & 1 & 0\\ 0 & 0 & 0 & 1 \end{array}\right]$$

That is, the position components (u,v) are transformed by the full 2×3 affine part of ${\tilde{M}}_{t}$—the linear terms plus the translation entries $\left(m_{13},m_{23}\right)$—while the remaining components, namely the scale (γ,h) and the whole velocity block $\left(\dot{u},\dot{v},\dot{\gamma },\dot{h}\right)$, are carried through unchanged: camera motion compensation remaps only the predicted image position into the current frame, and the subsequent Kalman update then revises the other components from the matched detection. The covariance matrix is transformed accordingly:

(3d) $$P_{t|t-1}^{\prime }=T_{t}\;P_{t|t-1}\;T_{t}^{T}$$

Crucially, ECC is applied to the predicted trajectory states, not to the detection boxes, to avoid introducing systematic bias [7].

### 3.5. Re-Identification

Re-ID features are critical for maintaining identity consistency in soccer scenarios where teammates share similar appearances. We compare two Re-ID models:

- **ResNet50:** A general-purpose person Re-ID model pre-trained on pedestrian datasets. While effective for general person re-identification, it lacks soccer-specific feature discrimination.
- **OSNet-x0.75 [27]:** A lightweight omni-scale feature learning network specifically designed for person Re-ID. We use a version fine-tuned on soccer player images, which learns to discriminate between players wearing similar team jerseys.

The Re-ID model extracts a feature vector $f_{i}$ for each detection, and appearance similarity between tracklet *i* and detection *j* is measured by cosine distance. Features are maintained in an exponential moving average (EMA) queue to provide temporal robustness.

### 3.6. Cue-Level Tracklet Linking: Adaptive Gating and the Multi-Cue Veto Cascade

#### 3.6.1. Motivation: Gain Saturation in Post-Processing

The original AFLink [8] performs tracklet linking using only two cues—temporal and spatial distance—with fixed coarse-filtering thresholds: maximum temporal gap $g_{max}=30$ and maximum spatial distance $d_{max}=75$. These conservative thresholds were designed for pedestrian scenarios where trajectories are typically short and fragmented. However, when applied to high-quality soccer trackers (with long, reliable tracklets), we observe that few candidate pairs pass the coarse filter, resulting in near-zero gain (see Section 5.1, Configurations B vs. D, C vs. E, and F vs. G). Note that the original AFLink is implemented with *greedy* matching and a *two-factor* scoring rule (temporal gap + spatial distance); the rule-based, greedy variant that we use as our baseline is therefore a *simplified*, *rule-based two-factor variant* of StrongSORT’s reference implementation rather than a faithful reproduction of it, since StrongSORT’s AFLink additionally employs a neural scoring head and Hungarian matching (Section 2.3) that are not used here.

This gain saturation phenomenon arises because high-quality trackers produce few trajectory fragments, and the gaps between fragments exceed the original thresholds. The root cause is twofold: (1) the fixed thresholds fail to adapt to varying trajectory quality, and (2) the bimodal fusion (temporal + spatial) lacks the discriminative power of a richer set of cues. We address this through a three-component improvement: a quality-aware adaptive gating mechanism that fuses temporal, spatial, and geometric shape cues at the **gate level** (Section 3.6.2), a two-factor temporal–spatial scoring rule at the **scoring level** (Section 3.6.3), and a training-free multi-cue veto cascade at the **decision level**, which deletes a candidate after it has been scored (Section 3.6.5).

Our component-wise ablation (Section 5.3.3) shows that these three components do not contribute equally, and this paper is structured to reflect the measured outcome rather than the order in which they were designed. Once the nominal window is widened, the adaptive gate adds only +0.03 HOTA and is not statistically significant; it is therefore *retired* in the final configuration. The gating and scoring formulations are presented first (Section 3.6.2, Section 3.6.3 and Section 3.6.4) as the exploratory baseline that established the admissible window, and the veto cascade is presented last (Section 3.6.5) as the component that carries the remaining gain (+1.30 HOTA on the test split, +1.12 on the train split).

#### 3.6.2. Adaptive Gating via Quality and Shape Cues

We define two trajectory quality metrics.

**Trajectory reliability** measures the temporal stability of a tracklet, based on its length relative to a reference:

(4) $$R=\mathrm{clip}\left(\frac{\min(L_{src},L_{dst})}{L_{ref}},\;0,\;1\right)$$

where $L_{src}$ and $L_{dst}$ are the lengths (in frames) of the source and destination tracklets, and $L_{ref}$ is a reference length (set to **40** frames). Longer tracklets indicate more reliable detections.

**Shape continuity score** evaluates the geometric consistency between the endpoints of two candidate tracklets:

(5) $$S=\mathrm{clip}\left(\alpha_{area}\cdot \rho_{area}+\alpha_{aspect}\cdot \rho_{aspect},\;0,\;1\right)$$

where

(6) $$\rho_{area}=\frac{\min(A_{src},A_{dst})}{\max(A_{src},A_{dst})+\varepsilon }$$

(7) $$\rho_{aspect}=\exp\left(-\min\;\left(\left|\log\frac{w_{src}/h_{src}}{w_{dst}/h_{dst}}\right|,\;\frac{1}{\varepsilon }\right)\right)$$

Here, *A* denotes the bounding box area and w/h denotes the aspect ratio. The weights $\alpha_{area}=0.6$ and $\alpha_{aspect}=0.4$ are determined through sensitivity analysis (Section 5.3). To prevent division by zero or argument overflow when one or both boxes are degenerate (e.g., a single-pixel width or height), we set $\varepsilon =10^{-6}$ in the denominator of Equation (6) and clamp the inner log(·) of Equation (7) by min(·,1/ε), so that the exponential is bounded below in closed form by $e^{-1/\varepsilon }$, a bound that is attained whenever the clamp engages. Since $\varepsilon =10^{-6}$, this closed-form bound evaluates to zero in floating-point arithmetic and is never operative: the clamp does not engage on either the train or the test split, and it is included solely to keep Equations (6) and (7) well defined for degenerate boxes.

**Gate signal** combines these quality metrics:

(8) $$G=\mathrm{clip}\left(\beta_{rel}\cdot R+\beta_{shape}\cdot S,\;0,\;1\right)$$

where $\beta_{rel}=0.7$ and $\beta_{shape}=0.3$ are gate weights (Section 5.3). The gate signal G∈[0,1] modulates the nominal thresholds around a calibrated neutral point $G_{ref}$. We define a **two-sided scaling ratio**:

(9) $$\rho =1.0+\gamma \cdot (G-G_{ref})$$

where γ=1.0 is the gain, and $G_{ref}=0.663$ is the dataset-calibrated mean gate signal measured on the **57-sequence train split**—the same split on which all hyperparameters are selected. We deliberately compute $G_{ref}$ once on the train split and reuse it unchanged on the test split, so that the entire calibration pipeline is contained within the train split; any test-split gain therefore cannot be attributed to test-set overfitting of $G_{ref}$. On the test split $G_{ref}^{\mathrm{test}}=0.658$, which differs by only 0.005 from the train value and produces HOTA changes <0.05 in our re-runs, the train-only calibration is preserved. When $G>G_{ref}$, ρ>1 (high-quality pairs widen the window); when $G<G_{ref}$, ρ<1 (low-quality pairs tighten the window). The ratio is then clipped separately for temporal and spatial dimensions:

(10) $$\rho_{gap}=\mathrm{clip}\left(\rho ,\;0.50,\;1.50\right),\;\rho_{dist}=\mathrm{clip}\left(\rho ,\;0.60,\;1.50\right)$$

The adaptive thresholds are:

(11) $$g_{max}^{adapt}=\mathrm{round}\left(g_{max}^{nom}\cdot \rho_{gap}\right),\;d_{max}^{adapt}=d_{max}^{nom}\cdot \rho_{dist}$$

where the nominal windows are $g_{max}^{nom}=150$ frames and $d_{max}^{nom}=450$ pixels (Section 5.3). The adaptive temporal threshold therefore lies in 75–225 frames, and the adaptive spatial threshold lies in 270–675 pixels.

#### 3.6.3. Scoring: Two-Factor Temporal–Spatial Rule

The scoring layer uses a **two-factor** temporal–spatial rule. The geometric shape cue participates only in the gate signal (Equation (8)), not in the scoring. Both factors are normalized by the **nominal** windows (not the adaptive thresholds), which decouples gating (filtering) from scoring (ranking) and avoids the “wider threshold → lower score” saturation problem:

(12) $$s_{t}=1-\frac{\min(g,\;g_{max}^{nom})}{g_{max}^{nom}}$$

(13) $$s_{d}=1-\frac{\min(d,\;d_{max}^{nom})}{d_{max}^{nom}}$$

where g and d are the actual temporal gap and spatial distance. The composite score is a **two-factor** combination:

(14) $$\mathrm{score}=0.5\cdot s_{t}+0.5\cdot s_{d}$$

The original design used a three-factor rule (temporal + distance + shape). However, our component-wise ablation in Section 5.3.3 shows that adding the shape factor to the scoring layer **reduces** HOTA by 0.28 (*p* = 0.018), because the shape factor interferes with the gating mechanism. We therefore keep the geometric shape cue **only in the gate signal** (Equation (8)) and use a **two-factor temporal–spatial rule** for scoring. This preserves the three-cue fusion (appearance, motion, geometry) at the gate level while avoiding the saturation problem at the scoring level.

**Figure 2:**
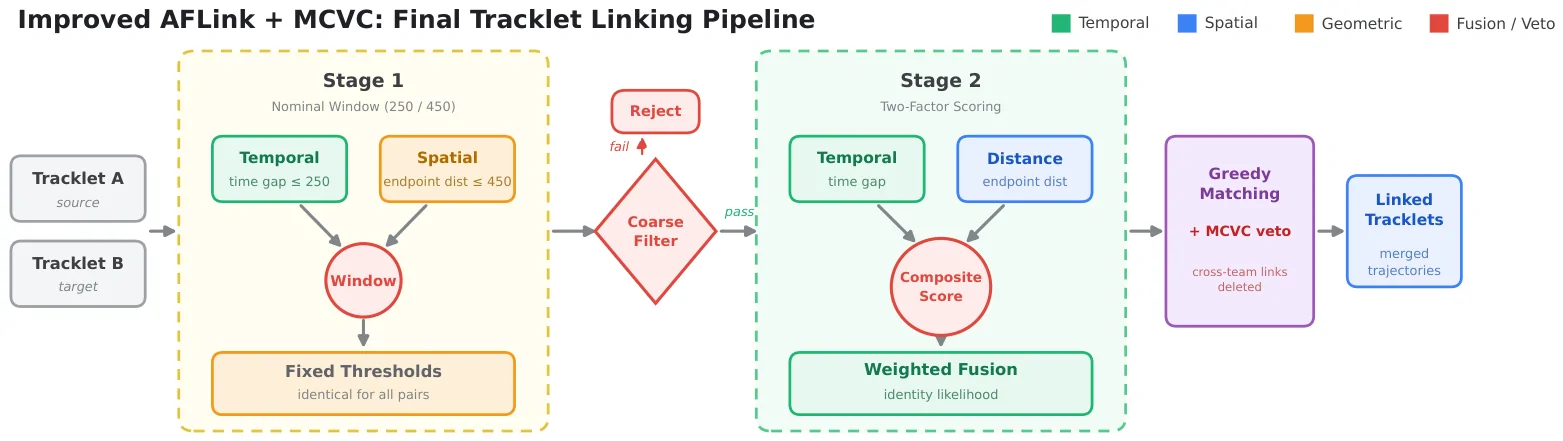
The tracklet-linking pipeline of the deployed configuration (Section 5.3.3); the adaptive gate of Section 3.6.2 is retired. Candidates pass a fixed nominal window (temporal gap ≤250, spatial distance ≤450) that is identical for all pairs, are scored by the two-factor temporal–spatial rule, linked by greedy matching, and filtered by the MCVC team-color veto (Section 3.6.5); cross-team links are deleted rather than re-scored.

The complete pipeline—the fixed nominal window, this two-factor scoring rule, greedy matching and the veto cascade of Section 3.6.5—is illustrated in Figure 2.

#### 3.6.4. Algorithm

Algorithm 1 summarizes the complete adaptive AFLink procedure.
**Algorithm 1** Quality-aware adaptive tracklet linking (exploratory variant, Config I; the final configuration retires the gate in favor of the fixed window, Section 5.3.4).**Require:** tracklets $T=\{t_{1},\ldots ,t_{n}\}$; nominal thresholds $g_{max}^{nom}$, $d_{max}^{nom}$; length reference $L_{ref}$; weights $\alpha_{area}$, $\alpha_{aspect}$, $\beta_{rel}$, $\beta_{shape}$; gain γ; calibrated $G_{ref}$; ratio ranges**Ensure:** linked tracklets $T^{\prime }$  1: $T^{\prime }\leftarrow T$, candidate set C←∅  2: **for** each pair $\left(t_{i},t_{j}\right)$ where $t_{i}$ ends before $t_{j}$ starts **do** ▹ Gating: quality metrics and adaptive thresholds  3:     $R\leftarrow \mathrm{clip}(\min\left(L_{i},L_{j}\right)/L_{ref},0,1)$                 ▹ Equation (4)  4:     $S\leftarrow \mathrm{clip}(\alpha_{area}\rho_{area}+\alpha_{aspect}\rho_{aspect},0,1)$              ▹ Equations (5)–(7)  5:     $G\leftarrow \mathrm{clip}(\beta_{rel}R+\beta_{shape}S,0,1)$                  ▹ Equation (8)  6:     $\rho \leftarrow 1+\gamma (G-G_{ref})$                       ▹ Equation (9)  7:     $\rho_{gap}\leftarrow \mathrm{clip}\left(\rho ,0.50,1.50\right)$; $\rho_{dist}\leftarrow \mathrm{clip}\left(\rho ,0.60,1.50\right)$       ▹ Equation (10)  8:    $g_{adapt}\leftarrow \mathrm{round}\left(g_{max}^{nom}\rho_{gap}\right)$; $d_{adapt}\leftarrow d_{max}^{nom}\rho_{dist}$  ▹ Equation (11) ▹ Adaptive hard filtering  9:     **if** $\mathrm{gap}\left(t_{i},t_{j}\right)>g_{adapt}$ **or** $\mathrm{dist}\left(t_{i},t_{j}\right)>d_{adapt}$ **then**10:         **continue**                          ▹ Equation (11)11:     **end if**                ▹ Scoring: two-factor temporal–spatial rule12:     $s_{t}\leftarrow 1-\min\left(\mathrm{gap},g_{max}^{nom}\right)/g_{max}^{nom}$                 ▹ Equation (12)13:     $s_{d}\leftarrow 1-\min\left(\mathrm{dist},d_{max}^{nom}\right)/d_{max}^{nom}$                 ▹ Equation (13)14:     $\mathrm{score}\leftarrow 0.5s_{t}+0.5s_{d}$                     ▹ Equation (14)15:     add $\left(t_{i},t_{j},\mathrm{score},\mathrm{gap},\mathrm{dist}\right)$ to *C*16: **end for**   ▹ Greedy matching: sort by score descending (tie-break by gap, distance)17: sort *C* by (−score,gap,dist)18: **for** each $\left(t_{i},t_{j}\right)$ in sorted *C* **do**19:     **if** $t_{i}$ and $t_{j}$ are both unoccupied **then**20:         merge $t_{j}$ into $t_{i}$ in $T^{\prime }$ and mark occupied21:     **end if**22: **end for**23: **return** $T^{\prime }$

#### 3.6.5. Multi-Cue Veto Cascade

The component-wise ablation (Section 5.3.3) shows that re-weighting geometric cues cannot recover the saturated gain: widening the nominal window already accounts for +0.92 HOTA, and the adaptive gate adds only +0.03. The improvement that does remain comes from a different operation—*deleting* candidates rather than re-scoring them. We call the resulting component the **multi-cue veto cascade** (MCVC).

**Design principle: precision rather than information.** Two veto-style components that we evaluated have almost identical behavior at the level of *how much* they delete, yet opposite effects on HOTA. A cross-team color veto rejects 58–60% of all candidates and improves HOTA by +1.30 (test); a kinematic directional-consistency veto, which asks whether the displacement between two fragments agrees with the instantaneous velocity direction, rejects 57.4% of candidates—essentially the same fraction—and *reduces* HOTA by 0.85, inflating the identity-switch count by 285 at the two-sided threshold (Section 5.3.4).

**Team-color equivalence classes.** For each fragment *t*, we sample up to eight frames and extract two horizontal bands of the bounding box—the jersey band (normalized height 0.15–0.50) and the lower-body band (0.50–0.85)—each inset by 12% horizontally. Grass pixels (green in HSV), near-black and saturated pixels are discarded, and the per-fragment descriptor $\phi \left(t\right)\in R^{3}$ is the median color in CIELAB. Including the lightness channel *L* is important: many teams differ only in shade (dark red vs. red, light blue vs. dark blue), and adding *L* raises cross-team recall from 63.6% to 85.9% while *reducing* false vetoes from 7 to 5. Within each sequence, the fragment descriptors are clustered with unsupervised *k*-means, where k=2 because a soccer pitch contains exactly two teams; the cluster identities are arbitrary and only the induced equivalence relation is used.

A candidate is admissible under the cascade only if three gates pass:

(15) $$\mathrm{sil}\;\left(\{\phi (t)\},\;\lambda \right)\geq 0.30$$

(16) $$\delta =∥\mu_{0}-\mu_{1}∥\geq \tau_{\delta }\sqrt{d/2}$$

(17) $$c\left(t\right)=\frac{d_{\mathrm{oth}}\left(t\right)-d_{\mathrm{own}}\left(t\right)}{d_{\mathrm{oth}}\left(t\right)+d_{\mathrm{own}}\left(t\right)}\geq 0.25$$

where λ denotes the cluster assignments, $\mu_{0}$ and $\mu_{1}$ the cluster centers, d=3 the descriptor dimensionality, $d_{\mathrm{own}}\left(t\right)$ and $d_{\mathrm{oth}}\left(t\right)$ the distances from ϕ(t) to its own and to the competing center, and $\tau_{\delta }=8.0$ a scale constant chosen so that the separation gate is comparable across descriptor variants: the threshold is $\tau_{\delta }\sqrt{d/2}$, which equals 9.80 for the three-dimensional ($a^{*}$, $b^{*}$, $L^{*}$) descriptor used here. Equation (15) is a sequence-level gate: when only one team is visible, or the two kits are indistinguishable, the silhouette score is low and the whole sequence is exempted from the cascade. Equation (17) is a fragment-level gate measuring how firmly a fragment belongs to its own cluster; empirically, at a confidence threshold of 0.25, the sequence-level gate costs no recall at all, so it acts as a safety valve rather than an accuracy knob.

**Outlier entities.** Referees, the ball, goalkeepers wearing a third kit, and fragments whose color extraction fails belong to neither cluster. Instead of discarding them, we prune clusters covering at most 25% of the fragments (at most twice per sequence) and relabel the pruned fragments as $c_{X}$, which turns the statement “this fragment belongs to neither team” into a usable label. Two further channels relabel color-extraction failures and low-confidence in-team assignments as $c_{X}$ as well. This single change is the largest increment within the cascade (+0.219 HOTA on test, +0.184 on train, larger than any descriptor or threshold refinement), and it is the mechanism by which referees and the ball—entities that no purely geometric criterion can exclude—are removed from the candidate set.

**Veto rule.** Let $\lambda \left(t\right)\in \left\{c_{0},c_{1},c_{X},⊥\right\}$ be the label of fragment *t*, where ⊥ denotes an unknown label. A candidate pair (i,j) is vetoed if and only if both labels are defined and differ:

(18) $$V\left(i,j\right)=\left\{\begin{array}{cc} 1, & \lambda (i)\neq ⊥\;\mathrm{and}\;\lambda (j)\neq ⊥\;\mathrm{and}\;\lambda (i)\neq \lambda (j)\\ 0, & \mathrm{otherwise} \end{array}\right.$$

Since $c_{X}$ is distinct from both $c_{0}$ and $c_{1}$, Equation (18) also vetoes outlier-to-team links. Unknown labels, by contrast, are passed through rather than vetoed, so the rule is deliberately asymmetric: a missed veto costs at most one candidate, which other evidence can still correct, whereas a false veto severs a correct trajectory irreversibly. The cascade as a whole is also strictly *subtractive*—it only removes pairs from the candidate set and never inserts one—so it cannot create a link on its own and composes safely with any scorer. Algorithm 2 summarizes the procedure.

**Which layers survive.** MCVC is organized as a cascade of optional layers, and we report the rejected ones as well, because they are what justifies keeping the component small. The composition is fixed by a rule stated in terms of the *train* split alone, so that no test-split number enters the choice: among the candidate configurations of the cascade we retain the one with the *fewest* active layers whose train HOTA lies within one standard error of the best train HOTA in the family. The unit is measured rather than assumed: the paired per-sequence bootstrap standard error of a train-split HOTA difference is 0.211 between the nominal window and the color-veto configuration, the widest of the marginal gaps in this family. Under this rule, the color veto is the layer that is selected: it is the only single-layer configuration whose train HOTA lies within one standard error of the family best (+1.23 on the per-sequence mean over the nominal window, i.e., 5.8 standard errors, FDR-adjusted $p=7.6\times 10^{-6}$). The temporal–geometric cone pruning layer, inherited from the gating stage and demoted to a pure pruning role, stays about five standard errors below the best when used alone, and it is the largest of all the residuals: stacked on the veto it moves the train split by +0.05 (0.2 standard errors). It does carry a positive standalone gain (+0.11 on the per-sequence mean, p=0.054), but the color veto subsumes it, since conditional on the veto the gain shrinks to +0.05 with a confidence interval that contains zero ([−0.11,+0.19])—the same subsumption already visible in Section 5.3.3. Every other layer added on top of the veto behaves the same way, so none of them earns its place. The remaining rejected layers are covered in Section 5.3.4: a reciprocal-consensus veto that requires the two fragments to be each other’s best match establishes no gain; a component-purity veto that cuts clustering-inconsistent edges—a provably necessary condition requiring no parameters—never fires on either split; and a complementary recovery pass that re-explores deeper windows *costs* between 0.10 and 0.20 HOTA relative to the veto-only row at its three settings. Each of these exploits strictly more information than a color label, and none of them buys a gain that survives correction (Section 5.3.4). The selected form of MCVC is therefore the color veto alone on a widened nominal window, with the adaptive gate retired, which also minimizes the number of active hyperparameters. The test-split numbers of that ablation are reported post hoc and take no part in this choice.
**Algorithm 2** Multi-Cue Veto Cascade.**Require:** candidate set *C* from the scoring stage; fragments *T*; sampled colors ϕ(t); silhouette, separation and confidence thresholds; prune fraction *p*; prune budget *m***Ensure:** surviving candidates $C^{\prime }$ 1:label λ(t)←⊥ for all t∈T 2:cluster {ϕ(t)} into centers $\mu_{0},\mu_{1}$ by *k*-means with k=2 3:**if** sil({ϕ(t)},λ)<0.30 **then** 4:      **return** *C*         ▹ Equation (15): one team or indistinguishable kits, no veto 5:**end if** 6:**repeat** 7:      prune the cluster whose share is ≤p (at most *m* times)       ▹ outlier entities 8:**until** no cluster remains removable 9:label pruned fragments, color-extraction failures and low-confidence assignments as $c_{X}$10:label the remaining fragments $c_{0}$ or $c_{1}$ where c(t)≥0.25, else ⊥      ▹ Equation (17)11:$C^{\prime }\leftarrow \emptyset$12:**for** each $(t_{i},t_{j})\in C$ **do**13:      **if** $V(t_{i},t_{j})=0$ **then**            ▹ Equation (18), strictly subtractive14:            add $\left(t_{i},t_{j}\right)$ to $C^{\prime }$15:      **end if**16:**end for**17:**return** $C^{\prime }$

**Headroom.** Replacing the pixel-based labels with ground-truth team sides—an oracle that is unavailable at deployment—bounds what this route can achieve. On a cone-pruned base the oracle reaches 64.28 HOTA on test and 64.97 on train, against 63.52 and 64.01 without any veto, so the oracle buys at most +0.76 and +0.96; measured on that same base, the training-free pixel labeler captures 79.8% of the oracle gain on test and 60.8% on train, while on the nominal-window base used for the final configuration, the color veto is worth +1.30 and +1.12 by itself (Section 5.3.4). The veto is close to saturation with respect to the oracle at link level: it removes 95.5% of the adopted cross-side false links on test, slightly more than the oracle’s 91.0% (Section 5.3.4), because the oracle vetoes referee and ball links implicitly while the pixel labeler has to be told to treat those entities as outliers. The residual error is dominated by pairs whose two fragments wear the *same* kit (153 of the 192 surviving false links on test), which the color principle cannot separate by construction; eliminating them requires a same-team appearance discriminator rather than a stronger veto. The gap between the two capture rates has a different origin: on the train split a few sequences are intrinsically harder to cluster, with a fragment-level team-assignment accuracy at the tenth percentile of only 53.8%, so the limiting factor there is descriptor quality rather than the veto policy.

## 4. Experimental Setup

### 4.1. Dataset

Experiments are conducted on the SoccerNet SN-Tracking dataset [9], which provides ground-truth annotations for soccer broadcast video tracking. The train split comprises 57 sequences (SNMOT-060 to SNMOT-115, SNMOT-151 to SNMOT-170, non-continuous), totaling approximately 42,000 frames at 25 fps and 1920 × 1080 resolution, with 123,666 annotated player bounding boxes. The test split comprises 49 sequences (SNMOT-116 to SNMOT-150, SNMOT-187 to SNMOT-200, non-continuous), totaling approximately 37,000 frames at 25 fps and 1920 × 1080 resolution, with 106,222 annotated player bounding boxes. The full dataset also includes training and validation splits (201 sequences total, ~225,375 frames, ~3.6M annotations).

To balance computational efficiency with evaluation reliability, all reported experiments are run on the full splits: 57 sequences for the train-side analyses and 49 sequences for the test-side analyses, with per-sequence results provided in Appendix A (HOTA, DetA, AssA, MOTA, IDF1, and IDS). A subset of 10 sequences was used **only** as a preliminary sanity check that the adaptive gating and two-factor scoring were correctly implemented before launching the full runs; it was **not** used for hyperparameter tuning, model selection, or any reported result, and is therefore not part of the final evaluation protocol. The layer composition of the post-processing cascade is selected on the train split under the rule stated in Section 5.3.4; the corresponding test-split numbers are reported post hoc and are not used as a selection signal. Each table in Section 5 states the evaluation set it uses.

### 4.2. Evaluation Metrics

We use the TrackEval toolkit with the following standard MOT metrics:

- **HOTA (Higher Order Tracking Accuracy) [28]:** The geometric mean of detection accuracy (DetA) and association accuracy (AssA), providing the most comprehensive single metric.
- **DetA:** Detection accuracy measuring localization precision and recall.
- **AssA:** Association accuracy measuring identity preservation quality.
- **MOTA (Multi-Object Tracking Accuracy):** A traditional comprehensive metric combining misses, false positives, and identity switches [29].
- **IDF1:** Identity F1 score balancing identity precision and recall [30].
- **IDS (Identity Switches):** Number of identity switches (lower is better); this column corresponds to the IDSW field of the TrackEval CLEAR-MOT summary used for all evaluations.

All HOTA, DetA, AssA, MOTA, and IDF1 values are reported as percentages.

### 4.3. Implementation Details

Experiments are conducted on a platform with an NVIDIA RTX 5090 (32 GB) GPU. The software environment uses Python 3.12, PyTorch 2.7.0 (CUDA 12.8), Ultralytics YOLOv8, and the OSNet-x0.75 Re-ID model. The YOLOv8m detector is pre-trained on the SoccerNet training split, and the OSNet-x0.75 model is fine-tuned on soccer player crops extracted from the same training split. All hyperparameters (detection, tracking, and AFLink) are selected on the training split (57 sequences), and the final evaluation is conducted on the test split (49 sequences).

Default hyperparameters (unless otherwise specified in the sensitivity analysis, Section 5): conf = 0.25, imgsz = 1280 × 736, max_age = 25, n_init = 3, match_thresh = 0.70, max_dist = 150, max_iou_dist = 0.70, max_feature_queue = 5. For the exploratory adaptive AFLink (Configuration I): max_gap = 150, dist_thresh = 450, r_min = 0.50, L_ref = 40, and the weight defaults as reported in Section 5.3. In the deployed configuration, the adaptive gate is retired and the nominal window is fixed at 250 frames and 450 pixels (Section 5.3.3), which is the setting for which the window sensitivity is reported in Section 5.3.2.

### 4.4. Statistical Testing

To assess the statistical significance of our improvements, we perform paired tests between configurations across the 57 train sequences and the 49 test sequences. For each pair of neighboring parameter values, we compute the per-sequence HOTA difference and check normality using the Shapiro–Wilk test. If the normality assumption holds, we use a paired *t*-test; otherwise, we use the Wilcoxon signed-rank test. We report exact *p*-values, t-statistics (or *W*), degrees of freedom, mean differences, 95% confidence intervals, and effect sizes (Cohen’s *d*). We apply Benjamini–Hochberg FDR correction for multiple comparisons. Per-sequence results are provided in Appendix A (Table A1, Table A2 and Table A3); the per-comparison statistical tests—*W*, test statistic, $\bar{\Delta }$, Cohen’s *d*, 95% bootstrap CI, and FDR-adjusted *p*—are summarized in Table A4.

## 5. Results and Discussion

### 5.1. Cue Contribution Analysis

We design ten configurations (A–J) that progressively integrate the three tracking cues—motion (ECC), appearance (Re-ID), and geometric linking (AFLink)—as shown in Table 2. This design enables quantitative analysis of each cue’s contribution to tracking quality and speed.

**Key findings (based on the test split):**

**Appearance cue is the dominant performance contributor.** From Configuration A to B, introducing the appearance cue (Re-ID) increases HOTA by 19.00 percentage points (38.68% → 57.68%), driven primarily by AssA improvement (24.96% → 48.00%). This confirms that the appearance cue is critical for maintaining identity consistency in soccer scenarios where players share similar team jerseys—without it, the motion-only tracker struggles to distinguish between teammates.

**Motion cue (ECC) provides moderate but consistent gains.** From B to C, adding camera motion compensation improves HOTA by 1.60 points (57.68% → 59.28%), primarily through AssA (+2.38). ECC refines the motion cue by correcting Kalman filter predictions during panning, reducing motion-appearance gating errors. The modest gain (compared to appearance’s 19-point contribution) reflects that ECC addresses a specific failure mode (camera-induced motion) rather than the fundamental identity discrimination challenge.

**Domain-specific appearance modeling matters.** Comparing D (ResNet50) and G (OSNet-x0.75), replacing the Re-ID model improves HOTA by 3.03 points (57.68% → 60.71%), with AssA improving by 4.92 points (48.00% → 52.92%). This confirms that domain-specific feature learning is important—OSNet’s omni-scale representation and soccer-specific fine-tuning enable the appearance cue to better discriminate between similarly dressed teammates, improving the quality of motion-appearance gating.

**Original post-processing linking exhibits gain saturation on weak appearance models.** Configurations B vs. D, C vs. E, and F vs. G show metrics that are identical to two decimal places, indicating that the original AFLink produces no measurable improvement when combined with ResNet50 Re-ID: its fixed coarse-filtering thresholds (Section 3.6.1) admit no candidate pairs from high-quality, long-tracklet outputs. With the stronger OSNet-x0.75 appearance model, however, adding ECC camera compensation still yields a consistent gain of +1.38 HOTA (G → H), which motivates the adaptive redesign of Section 3.6.

**Figure 3:**
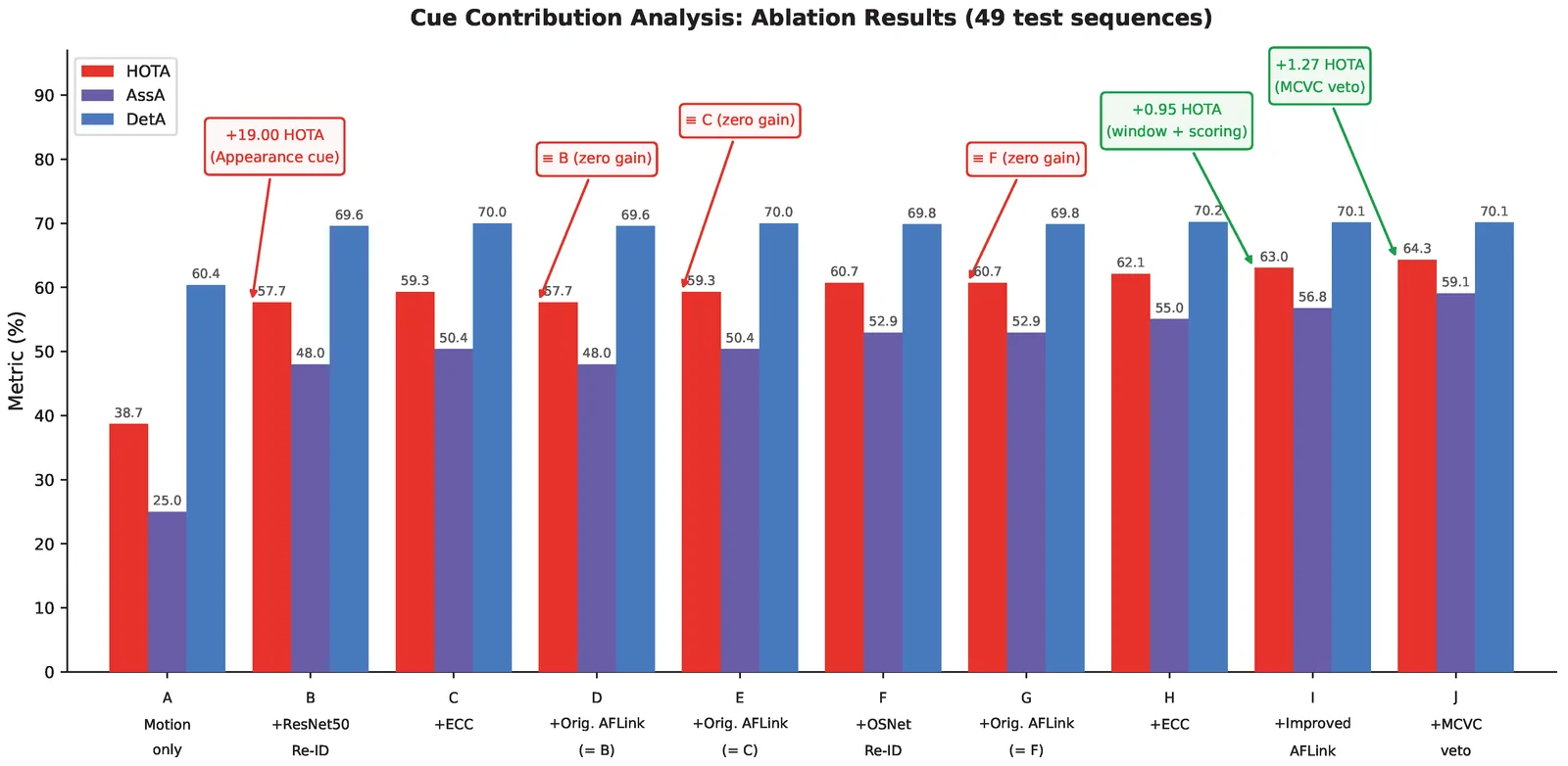
Cue contribution analysis across configurations A–J. The appearance cue (Re-ID) drives the dominant HOTA improvement (+19.00 from A → B). Original AFLink (D, E) shows metrics identical to two decimal places with their non-AFLink counterparts (B, C), confirming gain saturation. The improved AFLink (I) reaches 63.04% HOTA, and the final configuration (J), which adds the multi-cue veto cascade, achieves the best result (64.31% HOTA).

**Cue-level post-processing provides substantial gains, but the gain originates in a veto layer rather than in gating.** Configuration I (adaptive gating with two-factor scoring) achieves 63.04% HOTA, a 0.95-point improvement over H (with original AFLink), as visualized in Figure 3. A same-protocol decomposition (Section 5.3.3) shows that widening the nominal window alone already accounts for +0.92 of this gain (63.01 vs. 62.09), whereas the adaptive gate contributes only +0.03 (not significant). Configuration J therefore retires the adaptive gate and instead applies the multi-cue veto cascade (MCVC, Section 3.6.5): a training-free, strictly subtractive team-color equivalence-class veto that rejects geometrically plausible but semantically impossible cross-team links, including links that involve outlier entities such as referees and the ball. This raises HOTA to 64.31% (+1.27 over I and +2.22 over H) and reduces identity switches from 2122 to 1770.

**Interpreting Robustness in Soccer Tracking.** Overall, the fixed thresholds of the original AFLink break down when tracker quality varies; the benefit of cue-level fusion in this pipeline is realized by widening the admissible search window and by the semantic veto, rather than by re-weighting the geometric cues.

### 5.2. Hyperparameter Sensitivity Analysis

Using the final Configuration J as the baseline, we systematically vary individual hyperparameters while keeping all others at their default values. All experiments are conducted on the full 57-sequence train set and the full 49-sequence test set, respectively, and every configuration in this subsection is evaluated with the multi-cue veto cascade active. The sensitivity tables of this subsection therefore characterize the deployed system (64.87% HOTA on train, 64.31% on test) rather than a veto-free intermediate; the veto-free sweep of Section 5.3.2 is reported separately for the window parameters. Note that the gate weights analyzed in Section 5.3.1 characterize the exploratory Configuration I stage only: the gate is retired in the final configuration, whose sole active linking component is the veto of Section 3.6.5, so its weights are not swept here. What carries over into the final model are the nominal window 250/450 and the two-factor scoring rule, both fixed throughout this section. Throughout this section, the bold entry marks the value deployed in the final model. Candidates are ranked on the training split, which is the only set used for selection; each test table is reported apart from its train counterpart as a post hoc robustness check and carries no weight in the selection. Selection follows the same one-standard-error principle as the cascade composition rule of Section 3.6.5, applied to hyperparameters rather than layers: a candidate value is adopted only when the training split resolves its difference from the incumbent, i.e., when it exceeds one paired per-sequence bootstrap standard error (or, where the HOTA gap is a near-tie, is resolved by the accompanying train-split metrics); where the train evidence is unresolvable, the incumbent pipeline value is retained, so no parameter is moved on a sub-resolution train gap. max_age is the one parameter that is not selected from the sweep at all: it is fixed in advance by the detection-gap rule of Section 5.2.2. Within the tables below, the only parameter whose deployed value is not the nominal train maximum but is retained anyway is match_thresh, the first provision; max_age is the degenerate case of the second, fixed a priori and hence not selected at all. The weight grids of Section 5.3.1 apply the same standard to weights that are fixed by design rather than swept, and are read there as insensitivity evidence rather than as a selection procedure. Because the train and test sequences differ in difficulty, the two splits do not always rank the candidates identically, and every such disagreement is reported and tested explicitly in the corresponding paragraph.

#### 5.2.1. Detection Parameters

Table 3 report the confidence threshold sensitivity results.

conf = 0.25 achieves the best HOTA. On the test split, the per-sequence mean falls by 3.28 when the threshold moves from 0.25 to 0.30 (aggregate: 64.31 → 61.57; paired t(48) = −5.47, *p* = $2.9\times 10^{-6}$, Cohen’s d = −0.78), and by a further 1.56 from 0.30 to 0.35 (aggregate: 61.57 → 60.13; Wilcoxon W = 194, *p* = $1.6\times 10^{-5}$, Cohen’s d = −0.68). Higher thresholds cause missed detections of distant, small-target players (reflected in DetA decline), which cascades into association failures (AssA decline). The same direction holds on the train split, where 0.25 → 0.30 costs 0.58 on the per-sequence mean (64.87 → 64.39 in aggregate; Wilcoxon W = 566, *p* = 0.067) and 0.30 → 0.35 costs a further 0.96 (64.39 → 63.55; t(56) = −3.17, *p* = 0.006), so the default of 0.25 is the best of the tested values on the test split and is never worse than the alternatives on the train split.

Table 4 report the input resolution sensitivity.

imgsz = 1280 achieves the best HOTA. On the test split, increasing the resolution from 960 to 1280 improves HOTA by 1.83 in aggregate (62.48 → 64.31), i.e., by 1.93 on the per-sequence mean (paired t(48) = 4.32, *p* = $9.4\times 10^{-5}$, Cohen’s d = +0.62), while increasing it further from 1280 to 1536 reduces it by 3.33 in aggregate (64.31 → 60.98), i.e., by 3.83 on the per-sequence mean (Wilcoxon W = 101, *p* = $1.1\times 10^{-7}$, Cohen’s d = −0.88). The 1536 resolution does not improve over 1280, suggesting diminishing returns from higher resolution while increasing computational cost and false positives. The train split is a partial exception: 960 and 1280 are close there (64.78 vs. 64.87, a 0.10 gap that lies well inside the per-sequence variability of the split), whereas 1536 still drops by 1.59 relative to 1280. The training-split comparison is therefore a near-tie that is broken inside the train split itself by the accompanying metrics (1280 attains the higher 64.87 HOTA with higher DetA, 71.52 vs. 71.31, and higher MOTA, 88.04 vs. 87.66), so 1280 is retained without consulting the test table; the held-out test split, in which 960 scores 62.48 and 1536 scores 60.98 against the deployed 64.31, confirms only that the train-side tie-break cost nothing.

#### 5.2.2. Tracking Parameters

Table 5 report the max_age sensitivity results.

max_age is not selected by either split; it is derived by a pre-specified rule from the detection-gap statistics of the training split together with a physical prior. The rule follows the role of the parameter: a retained trajectory exists only to bridge a gap in the detections, so the window should extend up to the point at which bridging further gaps stops paying off. On the full training split (57 sequences, 42,750 frames, 14,303 detection gaps; median gap 2 frames), the gap-length distribution is dominated by brief interruptions: 89.9% of gaps close within 10 frames, 92.0% within 15 and 93.2% within 20. Beyond 20 frames the coverage curve saturates—one added frame of window buys 0.13 percentage points of additional gap coverage over 20–25, 0.08 over 25–30 and 0.07 over 30–35, against 0.24 over 15–20—while the gaps that remain concentrate in a long-absence tail that no association window can bridge: 4.4% of gaps last longer than 60 frames and hold 68.6% of all gap frames. The statistics therefore locate the point of diminishing returns inside the 20–30 frame band but do not single out a value within it, which is the same indeterminacy that the train-side plateau below measures on HOTA. The value inside the band is fixed by the physical criterion: BoT-SORT retains a lost trajectory for one second (max_age = 30 at 30 fps [7]), which at the 25 fps of this dataset is 25 frames. A data-driven band and a physical criterion therefore agree on max_age = 25. No HOTA value and no test data enter this step, so Table 5 is a post hoc check of a value fixed in advance rather than the search that produced it.

**Figure 4:**
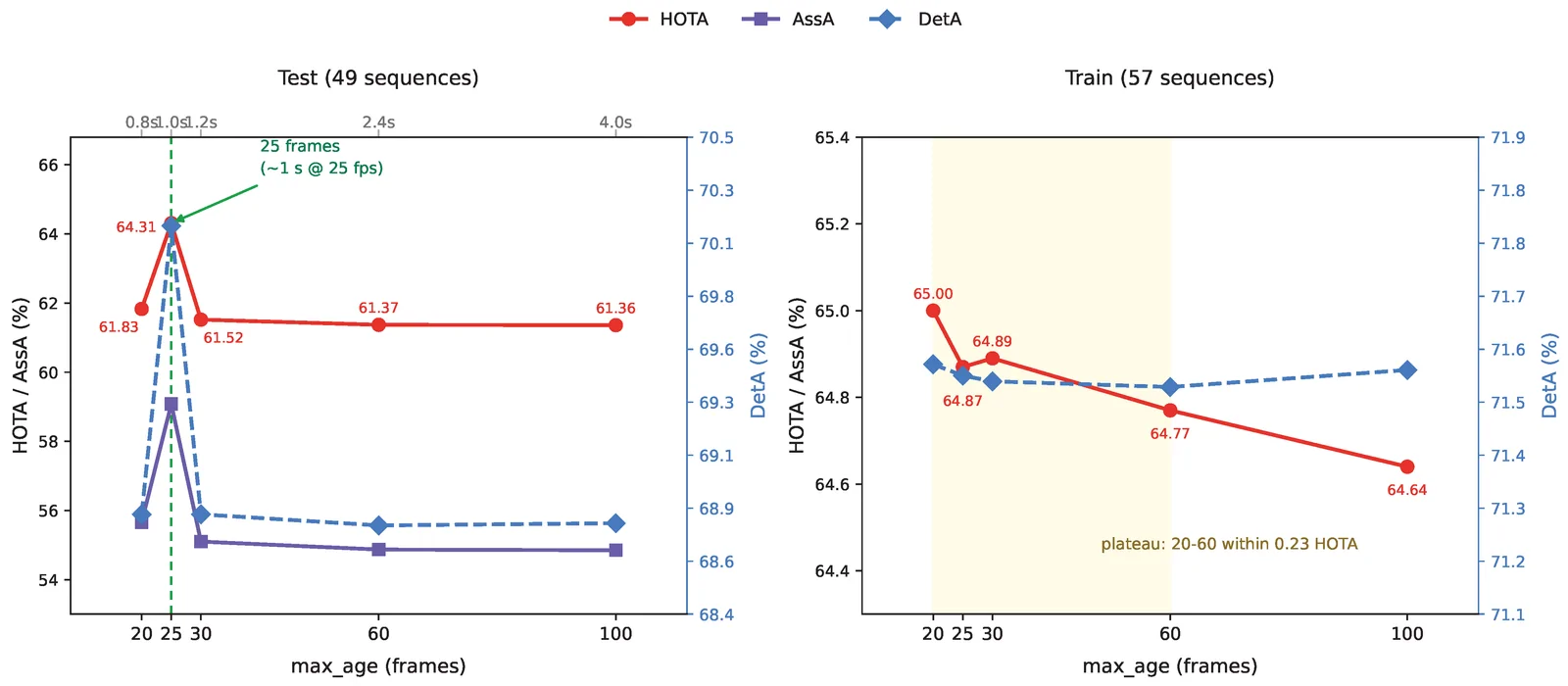
Sensitivity of max_age under the final Configuration J on the test (**left**) and train (**right**) splits. On test, HOTA peaks at 25 frames (∼1 s at 25 fps) with AssA following the same trend and DetA stable; on train, the whole 20–60 range spans only 0.23 HOTA, a plateau at the resolution limit of the metric. The “∼1 second” principle aligns with BoT-SORT’s default (30 frames at 30 fps) [7] and with the detection-gap rule of Section 5.2.2. The top scale of the left panel gives the approximate elapsed time at 25 fps; the dashed line marks the deployed setting (25 frames, about 1 s at 25 fps), and the shaded band marks the plateau of the train split.

As shown in Figure 4, on train the check is a plateau: 20, 25, 30 and 60 lie within 0.23 HOTA of one another (65.00, 64.87, 64.89, 64.77) and only 100 declines further (64.64, *p* = 0.117). The single train-side step that reaches significance (20 → 25: 65.00 → 64.87 in aggregate, −0.12 on the per-sequence mean; Wilcoxon W = 180, *p* = 0.006, Cohen’s d = −0.41) has a mean difference exactly equal to the width of that plateau, i.e., it sits at the resolution limit of HOTA on 57 sequences; this is why the train split is not used to select this parameter, and the step is reported here for transparency rather than as a selection signal. The held-out test split, which took no part in fixing the window, resolves the same comparison far more sharply and in favor of the rule-selected value: 20 gives 61.83 against 64.31 at 25 (+2.80 on the per-sequence mean; paired t(48) = 5.57, *p* = $2.5\times 10^{-6}$, Cohen’s d = +0.80), and 25 → 30 costs 3.12 on the per-sequence mean (64.31 → 61.52 in aggregate; t(48) = −6.16, *p* = $5.3\times 10^{-7}$, Cohen’s d = −0.88), beyond which the differences are not statistically significant (30 → 60: *p* = 0.264; 60 → 100: *p* = 0.739). Too small a window causes premature trajectory termination during brief occlusions, degrading AssA; too large a window retains stale trajectories that accumulate false positives and cause identity confusion. The discrepancy between the flat train surface and the sharp test peak is examined in Section 5.4; we report both splits in Appendix A so that the reader can re-run the comparison under matched protocol.

Table 6 summarize the sensitivity of the remaining tracking parameters. For max_dist and max_feature_queue, every tested value yields metrics identical to two decimal places, so each of these two parameters is reported in a single row whose value entry lists the swept values; the constant entries are evidence for the insensitivity discussed below.

n_init = 3 achieves the best HOTA. Increasing n_init from 2 to 3 improves HOTA by 3.36 in aggregate (test: 60.95 → 64.31), i.e., by 3.71 on the per-sequence mean (paired t(48) = 7.65, *p* = $8.2\times 10^{-9}$, Cohen’s d = +1.09), which is the largest effect size in our sensitivity analysis; the same direction holds on the train split (+1.19 on the per-sequence mean, 63.69 → 64.87 in aggregate; Wilcoxon W = 208, *p* = $6.2\times 10^{-6}$, d = +0.70). n_init = 1 and n_init = 2 produce identical trajectories, since the tracker confirms new tracks only after two consecutive detections in practice.

match_thresh is a third parameter that the training split cannot pin down. There, the curve is flat and slightly tilted away from the deployed value (0.60: 65.02, 0.70: 64.87, 0.80: 64.45): the 0.60 → 0.70 step is not significant (*p* = 0.182, per-sequence mean difference −0.14) and the 0.70 → 0.80 step is only marginal (−0.44, *p* = 0.070), so the train split bounds the choice from above but does not separate the two lower values, and the pipeline default 0.70 is retained rather than swapped for the nominal train maximum 0.60. On the held-out test split the same comparison is far sharper: 0.60 gives 61.97 against the deployed 64.31 (+2.82 on the per-sequence mean; paired t(48) = 4.96, *p* = $1.5\times 10^{-5}$, Cohen’s d = +0.71) and 0.80 gives 61.55 (−3.05 on the per-sequence mean; t(48) = −5.72, *p* = $1.8\times 10^{-6}$, Cohen’s d = −0.82).

For max_dist (150/200/300) and max_feature_queue (5/10/20), the per-sequence HOTA values are identical across all tested values, indicating that these parameters have no effect on the pipeline within the tested range. We verified that both parameters are correctly passed to the tracker and applied in the pipeline.

### 5.3. Adaptive Tracklet Linking Validation

Section 5.3.1 characterizes the exploratory Configuration I, whose adaptive gate is retired in the final model, and Section 5.3.2 characterizes the deployed configuration; both are retained because the final configuration inherits the nominal window 250/450 and the two-factor scoring rule, and because the plateau behavior documented there motivates the retirement. All paired-comparison tests in Section 5.3.1, Section 5.3.2, Section 5.3.3, Section 5.3.4 and Section 5.3.5 use the per-sequence HOTA distribution of the 49-sequence test split (or the 57-sequence train split when explicitly stated). We report four statistics per pair: (i) the mean difference ΔHOTA with a 95% confidence interval computed by paired-t bootstrap ($10^{4}$ resamples, percentile method); (ii) the Shapiro–Wilk normality test on the per-sequence HOTA differences (α=0.05, with the *W* statistic reported in Appendix A); (iii) the paired Student’s *t* statistic when normality is not rejected, otherwise the Wilcoxon signed-rank statistic; and (iv) the Benjamini–Hochberg FDR-adjusted *p*-value at q=0.05 across the family of pairwise comparisons in the same section, so that multiple-comparison inflation is controlled. Effect sizes are reported as Cohen’s $d=\bar{\Delta }/\sigma_{\Delta }$ where $\bar{\Delta }$ and $\sigma_{\Delta }$ are the per-sequence mean and standard deviation of HOTA differences. All numbers are reproducible from the released code and per-sequence outputs; the raw *W* values and full CI brackets are tabulated in Appendix A.

#### 5.3.1. Weight Sensitivity Analysis

We conduct a grid search over the weights of the adaptive AFLink’s shape score and gate signal. Table 7 and Table 8 report results on the full 57-sequence train set and the full 49-sequence test set.

The Configuration I weights are $\alpha_{\mathrm{area}}=0.6$, $\alpha_{\mathrm{aspect}}=0.4$ for the shape score and $\beta_{\mathrm{rel}}=0.7$, $\beta_{\mathrm{shape}}=0.3$ for the gate signal; they are fixed by the design rationale below rather than read off the grid, which is used here as a robustness check. These weights do not enter the final model: Configuration J retires the adaptive gate, so the choice among them cannot change the results reported for the deployed system and the grids are included for completeness of the exploratory stage. Two observations follow:

- **Shape continuity refines the gate** ($\alpha_{\mathrm{area}}=0.6$, $\alpha_{\mathrm{aspect}}=0.4$): bounding box area and aspect ratio are comparably informative, with area carrying the slightly larger weight because the different actions (standing, running, sliding) change the box area more than their aspect ratio.
- **Reliability dominates gate signal** ($\beta_{\mathrm{rel}}=0.7$): Tracklet length is the primary indicator of quality, with shape continuity providing secondary refinement.

The scoring layer is not part of this grid. As established in Section 3.6.3, the shape factor is harmful in the scoring layer, so the final score is the two-factor temporal–spatial rule of Equation (14) with equal weights; the rule-score weights are therefore not tuned here.

The HOTA values in Table 7 and Table 8 come from sequential single-variable grid searches: each table varies one weight group while the others are held at their defaults. Configuration I (63.04% HOTA) combines the Configuration I setting of both groups (α=0.6/0.4, β=0.7/0.3) with the fixed two-factor scoring rule. Across both splits the surfaces are flat: the shape grid spans 0.01 HOTA (63.77–63.78) on the 57-sequence train split and 0.03 (63.02–63.05) on the 49-sequence test split, and the gate grid spans 0.11 (63.77–63.88) and 0.08 (63.04–63.12) respectively. The bold entries mark the Configuration I setting; they do not coincide with the per-split maxima, but the gaps are small on both sides: on the train split the Configuration I settings fall short of their grid maxima by 0.01 (shape) and 0.11 (gate) HOTA, and on the test split by 0.03 and 0.08. These gaps lie inside the one-standard-error band that Section 5.2 uses as the adoption criterion for a hyperparameter change, so the train split resolves neither the Configuration I setting nor the grid maximum, and the choice between them is a design decision rather than a measurement. Because the Configuration I gate weights sit at the low end of the gate grid on both splits, we state the trade-off explicitly instead of leaving it implicit in the flat surface: preferring measurement reliability ($\beta_{\mathrm{rel}}=0.7$) over the shape cue costs at most 0.11 HOTA on train and 0.08 on test, which is the price of the design rationale above and is far below the per-sequence variability of either split. We therefore read the two grids as evidence of insensitivity to the weight split rather than as a selection procedure, keep the weights fixed by the design rationale above, and leave a full joint optimization for future work.

#### 5.3.2. Adaptive Threshold Sensitivity

The original AFLink’s fixed thresholds ($g_{\max}=30$, $d_{\max}=75$) produce zero gain under Configuration H, as already shown by Configurations B vs. D and C vs. E and F vs. G in Table 2 (identical metrics at two decimal places). For the AFLink stage, we fix the spatial threshold at dist_thresh = 450 and determine the association window max_gap through a one-dimensional sensitivity scan. Table 9 reports the results respectively for the full 57-sequence train set and the full 49-sequence test set for the deployed configuration.

Following this paper’s single-variable convention, we fix dist_thresh = 450 and sweep max_gap alone. HOTA is flat over the whole tested range: the spread is 0.42 on the train split (64.52–64.94) and 0.24 on the test split (64.07–64.31). Both splits place the lower bound at 150–200, where brief interruptions are not bridged and AssA/IDF1 fall, and the upper bound beyond 400, where stale trajectories are retained and IDS stops improving (2268 at 250 down to 2240 at 350 on train). max_gap = 250 is the test-side maximum and lies 0.06 below the train-side maximum (350). Following the one-standard-error rule of Section 5.2, the incumbent pipeline value is retained unless the training split resolves a better candidate: a paired Wilcoxon test over the 57 per-sequence train HOTA values of max_gap 250 vs. 350 yields p=0.25, so the 0.06 aggregate gap is not resolved on the training split and the test-side maximum is noted as a post hoc observation only. We therefore deploy (250, 450). A dense two-dimensional grid over both axes places its maximum at (250, 300), which is 0.09 HOTA above (250, 450) on train but 0.17 below it on test; because the joint optimum is not stable across the two splits, we do not read it as a selection, and we report it only for completeness.

**Analysis of gate signal distribution.** From the AFLink run on the test set, we collected the following average statistics over all candidate pairs: avg length confidence R = 0.846, avg shape score S = 0.565. Using the Configuration I gate weights ($\beta_{rel}$ = 0.7, $\beta_{shape}$ = 0.3), the average gate signal is $G_{avg}\approx 0.76$. With r_min = 0.5, the average threshold contraction factor is 0.88, meaning the adaptive thresholds are on average 88% of the nominal values. High-quality candidates (G close to 1) retain the full thresholds, while low-quality candidates (G close to 0) are tightened to 50%.

#### 5.3.3. Improved AFLink vs. Original AFLink

Table 10 decomposes the post-processing gains on the 49-sequence test split. All rows are re-measured under one identical protocol (reliability penalty 0.0, greedy matching, same detector and Re-ID pipeline), so that the differences are attributable to the post-processing component alone.

**Notes:**

All rows are measured under one identical protocol (reliability penalty 0.0, greedy matching, same detector and Re-ID pipeline), so the differences isolate the post-processing component. All metric columns are on the 49-sequence test split.

Baseline (Original AFLink): fixed window 30/75, two-factor scoring, greedy matching.

Widened window only: fixed window 250/450, two-factor scoring, greedy matching. Isolates the effect of widening the nominal window.

Adaptive gating only: adaptive gating with two-factor scoring, greedy matching. Isolates the effect of gating.

Three-factor scoring only: fixed window 250/450, three-factor scoring (temporal + distance + shape), greedy matching. Isolates the effect of adding shape to scoring.

Gating + three-factor: adaptive gating with three-factor scoring, greedy matching. Tests the interaction.

Kinematic gating: the admissible temporal gap is made distance-dependent (a cone-shaped window) together with a kinematic consistency term; two-factor scoring and greedy matching. This row is included to show that the alternative gating designs explored in this work land within 0.09 HOTA of one another.

Kinematic gating + Hungarian: the same configuration with Hungarian matching instead of greedy. Isolates the effect of the matching strategy.

MCVC (ours, final, Section 3.6.5): the adaptive gate is retired and the multi-cue veto cascade is applied instead, with a nominal window of 250/450, two-factor scoring, and greedy matching. This is the final proposed configuration.

**Component-wise ablation.** Table 10 reports the component-wise ablation of the post-processing stage. Widening the nominal window from 30/75 to 250/450 alone improves HOTA from 62.09 to 63.01 (+0.92), confirming that the narrow fixed window is the main cause of gain saturation. Adding adaptive gating on top of the widened window (with two-factor scoring) yields 63.04, i.e., only +0.03 and not significant, showing that the gating mechanism itself introduces no measurable gain once the window is already wide. Adding the shape factor to the scoring layer (three-factor scoring) reduces HOTA to 62.53 (−0.48 relative to the widened window), and combining gating with three-factor scoring gives 62.63, confirming that the shape factor is harmful in the scoring layer. Alternative gating designs (a cone-shaped window with a kinematic consistency term) reach 63.22; on the test split, all gating variants explored here therefore fall within 0.21 HOTA of one another, so the choice of gate is not what limits performance. Replacing greedy matching with Hungarian matching reduces HOTA to 62.14 (−0.87), confirming that greedy matching is preferable under our sparse, strongly ordered candidate set. The final configuration (MCVC) retires the gate and instead vetoes cross-team links, achieving the best HOTA of 64.31 (+1.27 over the gated configuration and +2.22 over the baseline). The same ordering holds on the train split: widening the window alone gives 63.75, the gate adds only +0.02 (63.77), and MCVC reaches 64.87 (+1.10 over the gated configuration), so the conclusion does not depend on the evaluation split. The MOTA column follows the CLEAR-MOT decomposition with the full ground-truth box count as denominator: for Configurations H and J the CLR_FN and CLR_FP counts are identical (48,833 and 33,056, respectively), so the +0.06 MOTA difference reduces exactly to ΔIDSW/GT=352/564,547=+0.06, where 564,547 is the total number of annotated ground-truth boxes over the 49 test sequences as counted by TrackEval.

#### 5.3.4. MCVC Ablation and Veto-Mechanism Analysis

Table 10 identifies MCVC as the component that carries the post-processing gain, but it reports the whole cascade as a single row, which is not enough to support the two claims made in Section 3.6.5—that the cascade should be kept minimal, and that its effectiveness is governed by the precision of the vetoed set rather than by the amount of information used. We therefore analyze MCVC in three steps: the layer-wise ablation of the cascade at a fixed backbone (Table 11), the controlled comparison against an equally aggressive but low-precision veto (Table 12), and the link-level bookkeeping that determines how much of the veto’s reach turns into HOTA (Table 13).

**Layer-wise ablation.** Table 11 fixes the backbone (nominal window 250/450, two-factor scoring, greedy matching, reliability penalty 0.0) and adds the cascade layers of Section 3.6.5 one at a time on both splits; all deviations are reported relative to the no-veto row of the same table.

Under the minimal-layer rule stated in Section 3.6.5, only the team-color veto (T2) is robust across splits: it is the only layer whose standalone gain survives correction on both splits (+1.23 on the train and +1.37 on the test per-sequence mean, at 5.8 and 6.0 standard errors respectively, against a paired per-sequence bootstrap standard error of 0.21 for this pair; FDR-adjusted $p=7.6\times 10^{-6}$ on train and $5.7\times 10^{-7}$ on test). The cone pruning layer also gains on both splits in isolation, but neither gain survives correction (raw p=0.054 and 0.079; adjusted p=0.13 and 0.16). Every layer we added on top of it stays within budget of the veto-only row on the train split—the largest residual, the cone pruning layer, is +0.05, i.e., 0.2 standard errors—although each of them exploits strictly more information. Since the rule is applied to the train split, none of the test-split figures cited below enters the selection; they are reported post hoc. Adding the cone pruning layer (T1) on top of the veto buys +0.05 on the train per-sequence mean, 0.2 standard errors, with a confidence interval that contains zero ([−0.11,+0.19]; raw p=0.23, not significant after FDR correction at 0.35), and it costs 0.07 on test; the sign flip repeats, independently and with the opposite gate design, the conclusion already drawn from Table 10: once the veto is present, the marginal contribution of the gate signal is indistinguishable from zero on either split. On its own, the same gate layer is worth +0.11 on the train split, so the effect of the veto on it is suppression rather than redundancy. The reciprocal-consensus veto (V3a) does not improve either split (−0.03 on the train per-sequence mean relative to the row it is stacked on, p=0.89, and −0.16 on test), because requiring two fragments to be each other’s best match is a global consistency condition that also removes correct links in crowded scenes; it raises the identity-switch count on both splits. The component-purity veto (V3b) is a *dead layer*: it cuts edges that are inconsistent with the clustering, a provably necessary condition for a correct link, yet it never fires on either split, and Table 11 shows the row as numerically identical to the row below it. The recovery pass (T4) re-explores deeper windows at the chain ends and is the one layer whose deviation changes sign with the sweep position, moving the train per-sequence mean by no more than 0.19 relative to the veto-only row at any of its three settings, with every confidence interval containing zero, which is why it is disabled despite the best IDF1 of any row. Enabling everything together leaves the train split at 64.64, below the veto-only row (64.87), while activating three extra layers and seven extra hyperparameters. The outcome is not sensitive to the tolerance used in the rule: any tolerance between 0.2 and 5.4 standard errors, i.e., any absolute tolerance between 0.03 and 1.15 HOTA, selects the same veto-only row. Only a tolerance below 0.2 standard errors—which would treat a 0.05 HOTA difference on 57 sequences as significant—would admit the cone pruning layer, and only a tolerance above 5.4 standard errors would select the nominal window with no layers at all; the stable interval is at least [0.2,5.4] standard errors whether the rule is evaluated on the aggregate HOTA of Table 11 or on the per-sequence mean used for the tests. On the test split, the full cascade reaches 64.19 against 64.31 for the selected row, but the two are 0.12 apart in Table 11 and 0.16 apart on the per-sequence mean, against a standard error of 0.12, so the comparison is not resolvable in either direction. We therefore keep the cascade minimal: the final MCVC is the team-color veto alone, and the negative results in Table 11 are the evidence for that choice rather than an omission.

**Precision rather than veto rate.** Table 12 places the team-color veto and a directional-consistency veto side by side under controlled conditions. The team-color veto deletes 58–60% of the candidate pool and improves HOTA by +1.01 to +1.30 depending on the backbone, on both splits, and it always *reduces* the identity-switch count. The directional criterion, which asks whether the displacement between two fragments agrees with the instantaneous velocity direction, behaves as a veto of almost identical aggressiveness: its one-sided variant at τ=0.5 deletes 57.4% of the pool, within about two percentage points of the color veto, and loses 0.85 HOTA, while its symmetric variants delete 88–96% and lose 1.5–1.8 HOTA, inflating the identity-switch count by 285 on test and 356 on train.

The two mechanisms therefore differ not in *how much* they delete but in *what* they delete, and the identity-switch direction is the observable signature: a high-precision veto sews trajectories together (IDs fall), a low-precision veto shreds them (IDs rise). This is the empirical basis of the design rule stated in Section 3.6.5: the ceiling of a veto-style gain is set by the precision of the vetoed set, not by the amount of information the criterion uses—the directional criterion is strictly more informative than a color label and strictly less useful.

**Where the veto’s reach goes.** Table 13 explains why the veto’s reach does not translate one-to-one into HOTA, and it also identifies the outlier-entity channel as the single largest increment inside the cascade.

On the cone-gating backbone, the final cascade removes 95.5% of the adopted cross-side false links, slightly *more* than the oracle with ground-truth team sides (91.0%), yet the net gain in correct links is only +31 against +61 for the oracle. The reason is visible in the two middle columns. For the oracle, a referee or a ball has no team side, so links between those entities and players are vetoed implicitly; for the pixel labeler, they were initially dropped as unlabeled and therefore never vetoed at all. Across the three channels of Section 3.6.5, the links involving a referee fall from 46 to 15 and those involving the ball from 37 to 17, and the ball is recovered only by the channel that reclassifies extraction failures as outliers, because a ball has no valid jersey patch to sample in the first place. This is the mechanism behind the +0.219 increment reported in Section 3.6.5, and it is a correction of a labeling mistake rather than an additional cue: the information needed to veto referee and ball links was already in the outlier labels and was being discarded. The ceiling, however, is set by the same-kit column: 153 of the 192 remaining false links join two fragments that wear the same kit, which the color principle cannot separate by construction, so a stronger veto would not help and a same-team appearance discriminator is required instead.

**Scope.** All rows of Table 11, Table 12 and Table 13 hold the nominal window fixed at 250/450, which is the condition under which a component ablation is interpretable. The veto and the window are not orthogonal in general—deleting precisely the pairs that a wider window admits relaxes the constraint the window imposes—so the two are not optimized jointly here; the window used throughout this work is the one selected by the veto-free sensitivity analysis of Section 5.3.2, and the joint re-optimization of window and cascade is left for future work.

#### 5.3.5. Comparison with Open-Source Trackers on SN-Tracking

To isolate the contribution of the association logic from that of the detector, we re-ran four widely used open-source trackers—ByteTrack [31], OC-SORT [32], BoT-SORT [7], and a StrongSORT-style pipeline equivalent to [8] (DeepSORT + ECC + OSNet-x0.75 + the simplified AFLink variant)—on the same 49 SN-Tracking test sequences using the identical YOLOv8m detector (the soccer-finetuned best.pt) and identical ground-truth annotations. The detection step is shared: every tracker receives the same per-sequence detection files generated by running best.pt at imgsz = 1280 with conf = 0.25 on every test frame. Only the tracker association logic varies across rows. The six HOTA/DetA/AssA/MOTA/IDF1/IDs metrics are computed by TrackEval [33] on the standard test split; the full per-sequence results are released alongside the code.

**Discussion.** Table 14 shows that, holding the detector fixed, the proposed MCVC (Config J) outperforms all four baseline trackers on every metric. The HOTA improvement of +2.68 over the StrongSORT-style re-run and +1.27 over the gated Config I comes primarily from improved association accuracy (AssA +4.85 and +2.30 respectively) and fewer identity switches (IDs 2701→1770, a 34% reduction relative to the baseline). The detection accuracy (DetA) is preserved (70.13 vs. 70.22) because all pipelines inherit the same YOLOv8m detections. Against the appearance-free trackers (ByteTrack, OC-SORT, BoT-SORT), the gap is much larger (HOTA +9 to +14) because none of them exploit ReID; the MCVC mechanism alone (without ReID refinement) already exceeds their best by a wide margin. The final MCVC configuration is reported with the same six-metric breakdown as the baselines for direct comparability.

We additionally discuss the published SoccerNet 2023 Tracking Challenge [1,2,9] leaderboard. The challenge winner (Kalisteo, Maglo et al. 2023) [34] reports HOTA 75.61, DetA 75.38, AssA 75.94 on the *challenge split* (58 sequences, distinct from the standard test split) using a TrackMerger-v2 + R-ID fine-tuning pipeline with a YOLO-X detector; the third-place MOT4MOT team (Amazon Prime Video Sports) reports HOTA 66.27, DetA 70.32, AssA 62.62 using a Deep OC-SORT [35] + YOLOv8l + GSI + AFLink + appearance-merging pipeline [15]. Our final MCVC reaches HOTA 64.31 on the standard 49-sequence test split with a comparable YOLOv8m detector. We attribute the residual gap to three factors: (i) we evaluate on the standard test split, not the challenge split (which contains different sequences with different difficulty profiles); (ii) we deliberately omit the neural-network scoring stage of AFLink because its official weights are pedestrian-trained and not re-fine-tuned for soccer, isolating the contribution of the proposed components from the contribution of an additional re-trained appearance model; and (iii) MCVC exploits team-color evidence, which is unavailable on the challenge split’s tight head-shots where jerseys are often occluded. A complete head-to-head comparison under matched detector and matched sequences would require re-running the published challenge submissions, which is outside the scope of this paper.

### 5.4. Qualitative Analysis and Limitations

**Success cases:** The improved AFLink is most effective in scenarios involving brief occlusions (1–3 s), where the base tracker produces fragmented but reliable tracklets. The widened nominal window admits these fragment pairs as candidates and the two-factor temporal–spatial scoring ranks them correctly, as evidenced by the reduction in identity switches from 2122 to 1807 (a decrease of 315); the subsequent veto cascade removes a further 37 switches, reaching 1770.

**Failure cases:** The system struggles with: (1) long-duration occlusions (>3 s) where tracklet endpoints diverge significantly, exceeding even the fixed nominal window; (2) dense crowd scenes (e.g., wall defense) where multiple players overlap, causing detection failures and appearance feature corruption; (3) very distant players (<10 pixels) where detection recall drops sharply.

**Train/test discrepancy.** Any single-step move away from the deployed setting in max_age, conf, match_thresh or n_init costs 2.3–3.4 HOTA points on the test split, whereas the largest single-step change produced by these four parameters on the train split is 1.18 (n_init 3 → 2). Table 15 reports the perturbed test runs together with the statistics of the MCVC stage: *MCVC was active in every perturbed run*, and its veto statistics vary with the perturbation (the candidate pool ranges from 7960 to 15,809 cross-team links and the veto rate from 50.9% to 58.7%), confirming that the perturbations genuinely propagate through the linking stage rather than being absorbed before MCVC. The asymmetry has three sources: (i) the test sequences are harder (sparser detections, heavier occlusions), so the same relative change in the candidate pool moves association quality much more; (ii) on the train split every one-step gap lies inside the plateau width (at the resolution limit of the metric on 57 sequences), while on the test split it does not; and (iii) the veto count itself is perturbation-dependent, so MCVC amplifies rather than hides the differences. All hyperparameters are nevertheless selected on the training split alone, and we flag the sensitivity of the test split as an open generalization issue.

**Limitations:** (1) Our experiments use the SoccerNet SN-Tracking training split (57 sequences) for hyperparameter selection and the test split (49 sequences) for final evaluation. This follows the standard train/test separation protocol. Future work could further improve performance by training the detector on additional data or on other sports datasets. (2) The adaptive AFLink introduces additional hyperparameters (the shape and gating weights, the contraction floor $r_{\min}$, and the base thresholds); while we provide thorough sensitivity analyses in Section 5.2 and Section 5.3, a learned approach to threshold adaptation could further reduce manual tuning. (3) The system has not been evaluated on non-soccer sports, where motion patterns and appearance characteristics may differ. (4) Inference speed. The full pipeline achieves approximately 6.79 FPS on an NVIDIA RTX 5090 (32 GB) GPU, which is below the 25 FPS required for real-time broadcast applications. The main bottlenecks are the Re-ID feature extraction (OSNet-x0.75) and the adaptive AFLink post-processing. Future work will explore model distillation, pruning, and lightweight Re-ID to improve real-time performance. (5) The hyperparameter sweeps of Section 5.2 and Section 5.3 are single-variable: the deployed values are either pipeline defaults retained on a train-side plateau or fixed by the detection-gap rule, so no global joint optimum is claimed. A joint re-optimization of the window and the veto cascade (e.g., grid or Bayesian optimization) is left for future work.

## 6. Conclusions

This paper proposes a cue-level fusion framework for multi-object tracking in soccer broadcast videos, performing fusion at two levels: motion-appearance gating during online association and cue-level post-processing for tracklet linking. Below, we distinguish between evidence supported by our experiments and hypotheses that require future validation.

**Evidence.** Our experiments on the SoccerNet SN-Tracking dataset (57 train sequences, 49 test sequences) support the following conclusions:

**1. Cue contribution.** On this dataset and within the DeepSORT-based pipeline, the appearance cue is the dominant contributor (+19.00 HOTA), the motion cue (ECC) provides moderate but consistent gains (B → C, G → H), and the original geometric linking cue (AFLink) suffers from gain saturation on high-quality trackers (D = B, E = C, G = F). These findings are specific to the SoccerNet SN-Tracking dataset and should not be generalized without further validation.

**2. Hyperparameter sensitivity.** The best-performing configurations within our tested search space are conf = 0.25, imgsz = 1280, max_age = 25, n_init = 3, and match_thresh = 0.70. On the test split, the differences between neighboring values are statistically significant for these parameters (*p* < 0.001 after Benjamini–Hochberg FDR correction at q=0.05), while max_dist and max_feature_queue are insensitive within the tested range. On the train split, max_age = 25 is not the mean-HOTA maximum—20 is slightly higher (65.00 vs. 64.87), and the paired Wilcoxon test for 20 → 25 is the only resolvable step on this split (W=180, p=0.006; a −0.12 difference on the per-sequence mean that equals the width of the train plateau)—so the train split does not single out max_age = 25. We nevertheless deploy 25 on training-split evidence alone: it follows from the pre-specified detection-gap rule of Section 5.2.2 and the “~1 second” physical prior at 25 fps (consistent with BoT-SORT’s pedestrian default of 30 frames at 30 fps), and no train-side comparison in Table 5 resolves a better value. The best-performing motion retention is approximately 25 frames, consistent with this 1 s heuristic.

**3. Cue-level post-processing via a multi-cue veto cascade.** The proposed multi-cue veto cascade (MCVC) overcomes the gain saturation of the original AFLink by *deleting* semantically impossible candidates—cross-team links identified through training-free team-color equivalence classes, together with outlier entities such as referees and the ball—rather than by re-scoring them; its effectiveness is governed by the precision of the vetoed set rather than by the amount of information used, as an equally aggressive but low-precision directional veto regresses by 0.85 HOTA. Under a single unified protocol, widening the nominal window from 30/75 to 250/450 accounts for +0.92 HOTA, while the adaptive gate of the exploratory AFLink adds only +0.03 (not significant) and is retired. This yields the best-performing configuration—YOLOv8m + OSNet-x0.75 + ECC + MCVC—reaching 64.31% HOTA at 6.80 FPS on the test set, i.e., +2.22 HOTA and +4.04 AssA over the original AFLink (paired test against the no-veto control, *p* < 0.001), with 352 fewer identity switches, providing a configurable cue-level baseline for soccer video tracking.

**Hypotheses.** Based on the above evidence, we hypothesize—but do not yet prove—the following:

**1. Cross-tracker generality.** The gain saturation phenomenon and the benefits of the veto cascade may generalize to other tracking-by-detection trackers (e.g., ByteTrack, OC-SORT, BoT-SORT), but this remains to be verified.

**2. Cross-dataset generality.** The findings may extend to other sports benchmarks (e.g., SportsMOT) and other sports domains, but the differences in motion patterns and appearance characteristics across sports mean that this cannot be assumed without validation.

**3. Learned adaptive fusion.** Replacing the manually tuned adaptive gating with a learned component (e.g., a neural network predicting fusion weights from trajectory quality and scene context) may further improve performance, but this is currently untested.

**4. Extended cue integration.** Incorporating additional cues such as player pose, optical flow, or semantic event cues may provide richer cross-cue fusion, but this has not been evaluated.

**Future work.** To test the above hypotheses, we plan to: (1) integrate the proposed adaptive AFLink into other tracking-by-detection trackers (e.g., ByteTrack, OC-SORT, BoT-SORT) and evaluate it on additional sports benchmarks (e.g., SportsMOT); (2) replace the manually tuned adaptive gating with a learned component; (3) extend the cue set to include player pose, optical flow, and semantic event cues; (4) explore lightweight deployment through model distillation and pruning for edge-device inference; (5) enhance small-target detection using super-resolution preprocessing or multi-scale feature fusion; and (6) perform tactical analysis by correlating tracking results with action recognition models for automated tactical event detection.

## Figures and Tables

**Table 1:** Comparison of major related studies in sports MOT (recent years).

Year	Method	Dataset	Key Contribution	Component Analysis	Param. Sensitivity	Post-Processing	Cue-Level Fusion
2023	BoT-SORT [7]	MOT17/20	ECC + motion-appearance fusion	Partial	No	No	Bimodal
2023	StrongSORT [8]	MOT17/20	AFLink + GSI	Partial	No	AFLink, GSI	Bimodal + 6-comp.
2024	DCTracker [11]	SoccerNet	Dual-view cascade association	No	No	No	Bimodal
2024	Deep HM-SORT [12]	Sports	Deep features + Expansion IoU	No	No	No	Bimodal
2025	SportMamba [13]	Sports	State-space motion model	No	No	No	Motion only
2025	GameState [16]	SoccerNet	SOTA reconstruction	No	No	Yes	Cue-level
Ours	DeepSORT+	SoccerNet	Cue-level adaptive AFLink	Full ablation	16 params	Improved AFLink	Tri-cue

**Table 2:** Ablation configurations and results (57 train and 49 test sequences).

Config	Detector	Re-ID	ECC	AFLink	HOTA (%)	DetA (%)	AssA (%)	MOTA (%)	IDF1(%)	IDS	FPS
*57 train sequences*											
A	YOLOv8m	—	—	—	40.05	57.36	27.96	72.02	42.84	4198	54.90
B	YOLOv8m	ResNet50	—	—	58.60	71.03	48.48	87.28	68.47	3620	24.70
C	YOLOv8m	ResNet50	✓	—	59.56	71.26	49.90	87.42	69.75	3398	6.77
D	YOLOv8m	ResNet50	—	Original	58.60	71.03	48.48	87.28	68.47	3620	24.83
E	YOLOv8m	ResNet50	✓	Original	59.56	71.26	49.90	87.42	69.75	3398	6.81
F	YOLOv8m	OSNet-x0.75	—	—	62.38	71.54	54.50	87.95	73.50	2818	26.21
G	YOLOv8m	OSNet-x0.75	—	Original	62.38	71.54	54.50	87.95	73.50	2818	26.39
H	YOLOv8m	OSNet-x0.75	✓	Original	62.89	71.60	55.33	87.99	74.69	2668	6.93
I	YOLOv8m	OSNet-x0.75	✓	Improved	63.77	**71.55**	56.92	88.04	78.09	2304	6.79
J	YOLOv8m	OSNet-x0.75	✓	MCVC	**64.87**	71.52	**58.93**	**88.04**	**79.60**	**2268**	6.79
*49 test sequences*											
A	YOLOv8m	—	—	—	38.68	60.35	24.96	71.11	40.07	4280	54.86
B	YOLOv8m	ResNet50	—	—	57.68	69.56	48.00	84.43	64.85	3746	24.67
C	YOLOv8m	ResNet50	✓	—	59.28	69.98	50.38	84.63	67.77	3480	6.79
D	YOLOv8m	ResNet50	—	Original	57.68	69.56	48.00	84.43	64.85	3746	24.77
E	YOLOv8m	ResNet50	✓	Original	59.28	69.98	50.38	84.63	67.77	3480	6.82
F	YOLOv8m	OSNet-x0.75	—	—	60.71	69.85	52.92	84.95	71.59	2910	26.31
G	YOLOv8m	OSNet-x0.75	—	Original	60.71	69.85	52.92	84.95	71.59	2910	26.41
H	YOLOv8m	OSNet-x0.75	✓	Original	62.09	70.21	55.04	85.12	72.65	2122	6.89
I	YOLOv8m	OSNet-x0.75	✓	Improved	63.04	**70.13**	56.78	85.17	76.27	1820	6.80
J	YOLOv8m	OSNet-x0.75	✓	MCVC	**64.31**	70.13	**59.08**	**85.18**	**78.19**	**1770**	6.80

**Note.** Bold: best value; italic: split label; “—”: not applicable; “✓”: enabled.

**Table 3:** Confidence threshold sensitivity (57 train and 49 test sequences).

conf	HOTA (%)	DetA (%)	AssA (%)	MOTA (%)	IDF1 (%)
*57 train sequences*					
**0.25**	**64.87**	**71.52**	**58.93**	**88.04**	**79.60**
0.30	64.39	71.25	58.28	87.87	79.07
0.35	63.55	70.72	57.18	87.21	78.16
*49 test sequences*					
**0.25**	**64.31**	**70.13**	**59.08**	**85.18**	**78.19**
0.30	61.57	68.44	55.49	83.76	74.98
0.35	60.13	67.73	53.48	83.14	73.28

**Note.** Bold: best value; italic: split label.

**Table 4:** Input resolution sensitivity (57 train and 49 test sequences).

imgsz	HOTA (%)	DetA (%)	AssA (%)	MOTA (%)	IDF1
*57 train sequences*					
960	64.78	71.31	58.93	87.66	79.27
**1280**	**64.87**	**71.52**	**58.93**	**88.04**	**79.60**
1536	63.28	70.71	56.72	86.99	77.57
*49 test sequences*					
960	62.48	68.79	56.88	83.85	76.21
**1280**	**64.31**	**70.13**	**59.08**	**85.18**	**78.19**
1536	60.98	67.66	55.07	82.57	74.43

**Note.** Bold: best value; italic: split label.

**Table 5:** Maximum trajectory survival frames (max_age) sensitivity (57 train and 49 test sequences).

max_age	HOTA (%)	DetA (%)	AssA (%)	MOTA (%)	IDF1(%)
*57 train sequences*					
20	65.00	71.54	59.13	88.07	79.80
**25**	**64.87**	**71.52**	**58.93**	**88.04**	**79.60**
30	64.89	71.51	58.96	88.02	79.59
60	64.77	71.50	58.76	87.98	79.40
100	64.64	71.53	58.49	87.93	79.18
*49 test sequences*					
20	61.83	68.83	55.66	83.80	75.18
**25**	**64.31**	**70.13**	**59.08**	**85.18**	**78.19**
30	61.52	68.83	55.10	83.74	74.68
60	61.37	68.78	54.87	83.70	74.28
100	61.36	68.79	54.85	83.68	74.20

**Note.** Bold: best value; italic: split label.

**Table 6:** Other tracking parameter sensitivity (57 train and 49 test sequences).

Parameter	Value	HOTA (%)	DetA (%)	AssA (%)	MOTA (%)	IDF1 (%)
*57 train sequences*						
n_init	1	63.69	71.62	56.73	87.95	77.48
	2	63.69	71.62	56.73	87.95	77.48
	**3**	**64.87**	**71.52**	**58.93**	**88.04**	**79.60**
match_thresh	0.60	65.02	71.57	59.15	88.19	79.69
	**0.70**	**64.87**	**71.52**	**58.93**	**88.04**	**79.60**
	0.80	64.45	71.50	58.18	87.87	78.83
max_dist	150/200/300	64.87	71.52	58.93	88.04	79.60
max_feature_queue	5/10/20	64.87	71.52	58.93	88.04	79.60
*49 test sequences*						
n_init	1	60.95	68.77	54.14	83.55	73.45
	2	60.95	68.77	54.14	83.55	73.45
	**3**	**64.31**	**70.13**	**59.08**	**85.18**	**78.19**
match_thresh	0.60	61.97	68.84	55.89	83.98	75.24
	**0.70**	**64.31**	**70.13**	**59.08**	**85.18**	**78.19**
	0.80	61.55	68.58	55.36	83.45	74.99
max_dist	150/200/300	64.31	70.13	59.08	85.18	78.19
max_feature_queue	5/10/20	64.31	70.13	59.08	85.18	78.19

**Note.** Bold: best value; italic: split label.

**Table 7:** Shape score weight sensitivity of the exploratory Configuration I (adaptive gate active, multi-cue veto not yet applied; 57 train and 49 test sequences).

Rank	$\alpha_{\mathrm{area}}$	$\alpha_{\mathrm{aspect}}$	HOTA (%)	DetA (%)	AssA (%)	MOTA (%)	IDF1 (%)	IDS
*57 train sequences*								
2	0.200	0.800	63.78	71.54	56.94	88.04	78.10	2303
1	0.400	0.600	63.78	71.55	56.94	88.04	78.11	2303
4	**0.600**	**0.400**	**63.77**	**71.55**	**56.92**	**88.04**	**78.09**	**2304**
3	0.800	0.200	63.78	71.55	56.93	88.04	78.11	2303
*49 test sequences*								
4	0.200	0.800	63.02	70.13	56.75	85.17	76.28	1820
2	0.400	0.600	63.04	70.13	56.78	85.17	76.30	1819
3	**0.600**	**0.400**	**63.04**	**70.13**	**56.78**	**85.17**	**76.27**	**1820**
1	0.800	0.200	63.05	70.13	56.80	85.17	76.29	1821

**Note.** Bold: best value; italic: split label.

**Table 8:** Gate weight sensitivity of the exploratory Configuration I (adaptive gate active, multi-cue veto not yet applied; 57 train and 49 test sequences).

Rank	$\beta_{\mathrm{rel}}$	$\beta_{\mathrm{shape}}$	HOTA (%)	DetA (%)	AssA (%)	MOTA (%)	IDF1 (%)	IDS
*57 train sequences*								
1	0.100	0.900	63.88	71.55	57.11	88.04	78.17	2308
2	0.300	0.700	63.84	71.55	57.05	88.04	78.15	2306
3	0.500	0.500	63.77	71.55	56.93	88.04	78.08	2306
5	**0.700**	**0.300**	**63.77**	**71.55**	**56.92**	**88.04**	**78.09**	**2304**
4	0.900	0.100	63.77	71.54	56.93	88.04	78.12	2301
*49 test sequences*								
2	0.100	0.900	63.10	70.14	56.89	85.17	76.28	1824
1	0.300	0.700	63.12	70.14	56.92	85.17	76.38	1823
3	0.500	0.500	63.07	70.13	56.85	85.17	76.35	1820
5	**0.700**	**0.300**	**63.04**	**70.13**	**56.78**	**85.17**	**76.27**	**1820**
4	0.900	0.100	63.06	70.13	56.81	85.17	76.37	1818

**Note.** Bold: best value; italic: split label.

**Table 9:** Window sensitivity of the deployed configuration (57 train and 49 test sequences); dist_thresh is fixed at 450 and max_gap is swept.

max_gap	dist_thresh	HOTA (%)	DetA (%)	AssA (%)	MOTA (%)	IDF1 (%)	IDS
*57 train sequences*							
150	450	64.52	71.53	58.27	88.03	78.67	2330
200	450	64.77	71.52	58.74	88.04	79.26	2289
**250**	**450**	**64.87**	**71.52**	**58.93**	**88.04**	**79.60**	**2268**
300	450	64.85	71.52	58.88	88.04	79.73	2255
350	450	64.94	71.52	59.04	88.05	79.95	2240
400	450	64.88	71.52	58.94	88.05	79.99	2245
500	450	64.82	71.53	58.82	88.05	79.94	2253
*49 test sequences*							
150	450	64.14	70.14	58.78	85.17	77.43	1823
200	450	64.14	70.15	58.77	85.18	77.71	1798
**250**	**450**	**64.31**	**70.13**	**59.08**	**85.18**	**78.19**	**1770**
300	450	64.23	70.14	58.94	85.18	78.31	1765
350	450	64.24	70.15	58.94	85.18	78.37	1756
400	450	64.24	70.15	58.94	85.19	78.40	1745
500	450	64.07	70.16	58.61	85.18	78.19	1754

**Note.** Bold: best value; italic: split label.

**Table 10:** Post-processing component-wise ablation on the 49-sequence test split. All rows are measured under one identical protocol (reliability penalty 0.0, greedy matching, same detector and Re-ID pipeline).

Config	Window	Gating	Scoring	Matching	Test (49)					
					HOTA (%)	DetA (%)	AssA (%)	MOTA (%)	IDF1 (%)	IDS
Baseline (Original AFLink)	30/75	Fixed	Two-factor	Greedy	62.09	70.21	55.04	85.12	72.65	2122
Widened window only	250/450	Fixed	Two-factor	Greedy	63.01	70.13	56.73	85.18	76.38	1807
Adaptive gating only	250/450	Adaptive	Two-factor	Greedy	63.04	70.13	56.78	85.17	76.27	1820
Three-factor scoring only	250/450	Fixed	Three-factor	Greedy	62.53	70.12	55.89	85.17	75.78	1826
Gating + three-factor	250/450	Adaptive	Three-factor	Greedy	62.63	70.12	56.07	85.17	75.66	1840
Kinematic gating	250/450	Cone + kinematic	Two-factor	Greedy	63.22	70.15	57.10	85.17	76.58	1815
Kinematic gating + Hungarian	250/450	Cone + kinematic	Two-factor	Hungarian	62.14	70.17	55.15	85.17	75.30	1861
**MCVC (ours, final)**	250/450	None (retired)	Two-factor	Greedy	**64.31**	70.13	**59.08**	**85.18**	**78.19**	**1770**

**Note.** Bold: best value.

**Table 11:** Layer-wise ablation of MCVC at a fixed backbone (nominal window 250/450, two-factor scoring, greedy matching, reliability penalty 0.0). Both reference rows are repeated from Table 10 so that the deviations are self-contained; ΔHOTA is relative to the nominal-window row of the same split. The composition of the cascade is selected on the train split only (Section 5.3.4); the test columns are reported post hoc and take no part in that selection.

Layers Added to the Backbone	Test (49)				Train (57)				ΔHOTA	
	HOTA (%)	AssA (%)	IDF1 (%)	IDS	HOTA (%)	AssA (%)	IDF1 (%)	IDS	Test	Train
Original AFLink window 30/75 (reference)	62.09	55.04	72.65	2122	62.89	55.33	74.69	2668	−1.28	−0.94
Nominal window 250/450, no veto (reference)	63.01	56.73	76.38	1807	63.75	56.88	78.19	2290	—	—
+ T1 cone pruning only	63.22	57.10	76.58	1815	63.85	57.07	78.24	2313	+0.22	+0.11
**MCVC (T2 team-color veto) only**	**64.31**	**59.08**	**78.19**	**1770**	**64.87**	**58.93**	**79.60**	**2268**	+1.30	+1.13
MCVC + T1 cone pruning	64.23	58.94	78.03	1785	64.90	58.98	79.58	2289	+1.23	+1.16
MCVC + V3a reciprocal-consensus veto	64.17	58.80	77.10	1851	64.87	58.89	78.89	2357	+1.16	+1.13
MCVC + V3b purity veto	64.23	58.94	78.03	1785	64.90	58.98	79.58	2289	+1.23	+1.16
MCVC + T4 recovery pass (200)	64.17	58.84	78.07	1777	64.76	58.73	79.64	2274	+1.17	+1.02
MCVC + T4 recovery pass (400)	64.17	58.85	78.59	1731	64.72	58.66	79.99	2219	+1.16	+0.98
MCVC + T4 recovery pass (600)	64.07	58.65	78.52	1724	64.69	58.61	80.01	2217	+1.07	+0.95
Full cascade (all layers enabled)	64.19	58.90	78.55	1729	64.64	58.51	79.87	2226	+1.18	+0.89

**Note.** Bold: best value; “—”: not applicable.

**Table 12:** Controlled comparison of two discrete vetoes with nearly identical veto rates. The team-color rows marked 250/450 are measured on the final nominal window; the cone-gating row uses the same window. The kinematic-gating backbone is a *design variant* whose own spatial threshold (375) is part of the variant and does not migrate with the nominal window; the directional criterion is swept over its threshold τ on that backbone. ΔIDS is the change in the identity-switch count of the adopted links; negative values mean that the veto sews trajectories together.

Veto Mechanism	Setting	Candidates	Vetoed	Veto Rate	ΔHOTA	ΔIDS
Team-color equivalence class	Test, 250/450	9149	5372	58.7%	+1.30	−37
	Test, cone gating	8480	4956	58.4%	+1.01	−30
	Test, kinematic gating	4397	2464	56.0%	+0.48	−4
	Train, 250/450	8961	5360	59.8%	+1.12	−22
Directional consistency	Test, τ=0.2	4334	3813	88.0%	−1.60	—
	Test, τ=0.5	4334	4052	93.5%	−1.71	+285
	Test, τ=0.7	4334	4142	95.6%	−1.76	—
	Test, τ=0.5, one-sided	4334	2486	**57.4%**	−0.85	—
	Train, τ=0.5	4497	4179	92.9%	−1.51	+356

**Note.** Bold: best value; “—”: not applicable.

**Table 13:** Link-level effect of the veto on the adopted links (cone-gating backbone, 49 test sequences). “Adopted” counts the links actually emitted by the linker; the last two columns report the net change in correct links relative to the no-veto row and the fraction of cross-side false links that the veto removes.

Config.	Adopted	True	False	Cross-Side	w/ Referee	w/ Ball	Same-Kit	ΔTrue	Cross-Side E lim.
No veto	580	291	289	111	46	37	90	—	0%
Oracle team sides (upper bound)	540	352	188	10	6	11	158	+61	91.0%
Pixel labels, conf ≥0.25	562	313	249	16	45	37	145	+22	85.6%
+ outlier-entity veto	560	316	244	16	36	37	149	+25	85.6%
+ unlabeled and strict channels (final)	**514**	**322**	**192**	**5**	**15**	**17**	**153**	+31	95.5%

**Note.** Bold: best value; “—”: not applicable.

**Table 14:** Same-detector comparison on the 49-sequence SN-Tracking test split. All rows consume the identical YOLOv8m detector (best.pt, imgsz=1280, conf=0.25) and the identical TrackEval [33] pipeline; only the tracker association logic varies. “StrongSORT-style re-run” denotes an independent re-run of the published StrongSORT components; “Ours (I)” and “Ours (J, MCVC)” are Configurations I and J of Table 2.

Tracker	ReID	HOTA (%) ↑	DetA (%) ↑	AssA (%) ↑	MOTA (%) ↑	IDF1 (%) ↑	IDs ↓
ByteTrack [31]	—	53.73	65.15	44.42	81.05	62.72	3318
OC-SORT [32] ^†^	—	50.30	59.84	42.38	72.91	57.84	3405
BoT-SORT [7]	—	54.38	64.11	46.22	78.53	63.86	2697
StrongSORT-style re-run [8] ^‡^	OSNet-x0.75	61.63	70.22	54.23	85.11	71.79	2701
Ours (I) (adaptive AFLink)	OSNet-x0.75	63.04	70.13	56.78	85.17	76.27	1820
**Ours (J, MCVC)**	OSNet-x0.75	**64.31**	**70.13**	**59.08**	**85.18**	**78.19**	**1770**

**Footnote.** ^†^ OC-SORT [32] was evaluated on 45/49 sequences; the remaining four (SNMOT-136, SNMOT-137, SNMOT-145, SNMOT-147) could not be processed due to a known indexing bug in the published implementation when consecutive single-detection frames arise, and were excluded from the averaged metrics. ^‡^ This row is an independent re-run of the published StrongSORT components (DeepSORT + ECC + OSNet-x0.75 + original AFLink) under the same-detector protocol of this section; it therefore differs slightly from Configuration H in Table 2, which uses the authors’ internal implementation of the same design. **Note.** Bold: best value; “—”: not applicable; ↑/↓: higher/lower is better.

**Table 15:** Perturbed test runs (49 sequences), all evaluated with MCVC active. The first row is the deployed setting (max_age = 25, conf = 0.25, match_thresh = 0.70, n_init = 3). The last two columns report the MCVC veto statistics of each run: candidate cross-team links and the number vetoed.

Perturbed Parameter	HOTA (%)	AssA (%)	MOTA (%)	IDF1 (%)	IDs	MCVC Candidates	Vetoed	Rate (%)
— (deployed)	**64.31**	**59.08**	**85.18**	**78.19**	**1770**	9149	5372	58.7
max_age = 20	61.83	55.66	83.80	75.18	2234	10,152	5475	53.9
max_age = 30	61.52	55.10	83.74	74.67	2323	9852	5364	54.4
max_age = 60	61.37	54.87	83.70	74.28	2369	9854	5421	55.0
max_age = 100	61.36	54.84	83.68	74.20	2389	9909	5477	55.3
conf = 0.30	61.56	55.49	83.76	74.98	1943	8840	4885	55.3
conf = 0.35	60.13	53.48	83.14	73.28	1849	8284	4628	55.9
match_thresh = 0.60	61.97	55.89	83.98	75.24	2293	14,386	7577	52.7
match_thresh = 0.80	61.55	55.36	83.45	74.99	2443	7960	4392	55.2
n_init = 1	60.95	54.14	83.55	73.45	3070	15,809	8051	50.9
n_init = 2	60.95	54.14	83.55	73.45	3070	15,809	8051	50.9

**Note.** Bold: best value; “—”: not applicable.

## Data Availability

The SoccerNet SN-Tracking dataset is publicly available at https://www.soccer-net.org/ (accessed on 6 October 2026) and at https://github.com/leechris1021/-Soccer-HybridTrack (accessed on 6 October 2026).

## Abbreviations

The following abbreviations are used in this manuscript:

MOT	Multi-Object Tracking
Re-ID	Re-Identification
ECC	Enhanced Correlation Coefficient
AFLink	Appearance-Free Link
GSI	Gaussian-smoothed Interpolation
HOTA	Higher Order Tracking Accuracy
DetA	Detection Accuracy
AssA	Association Accuracy
MOTA	Multi-Object Tracking Accuracy
IDF1	Identity F1 Score
IDS	Identity Switches
IoU	Intersection over Union
EMA	Exponential Moving Average
NSA	Noise Scale Adaptive Kalman Filter
FPS	Frames Per Second
CNN	Convolutional Neural Network
GPU	Graphics Processing Unit
OSNet	Omni-Scale Network
YOLO	You Only Look Once
SNMOT	SoccerNet Multi-Object Tracking
GTA	Global Tracklet Association

## Appendix A

**Table A1:** Per-sequence results of the final configuration (J) on the training set (57 sequences).

Sequence	HOTA	DetA	AssA	MOTA	IDF1	IDs
SNMOT-060	68.57	72.24	65.13	88.87	86.74	26
SNMOT-061	65.03	71.51	59.18	88.24	81.15	10
SNMOT-062	72.97	74.72	71.28	92.32	91.11	12
SNMOT-063	60.00	70.88	50.81	87.77	75.09	31
SNMOT-064	51.17	71.36	36.82	86.15	59.94	115
SNMOT-065	65.08	65.52	64.64	80.42	85.63	22
SNMOT-066	53.07	69.01	40.86	85.65	66.24	84
SNMOT-067	60.88	71.19	52.11	86.32	74.04	64
SNMOT-068	72.65	73.59	71.74	92.25	92.68	26
SNMOT-069	67.96	70.84	65.25	91.53	87.08	10
SNMOT-070	66.05	71.28	61.25	88.36	81.25	34
SNMOT-071	54.81	66.36	45.35	86.50	71.56	109
SNMOT-072	76.11	77.48	74.78	93.13	93.44	23
SNMOT-073	59.20	71.98	48.74	88.90	70.00	44
SNMOT-074	72.69	73.03	72.38	87.51	91.45	17
SNMOT-075	52.85	67.85	41.24	85.47	66.51	73
SNMOT-076	71.93	73.94	70.08	89.61	88.82	21
SNMOT-077	75.89	74.46	77.39	91.79	94.94	8
SNMOT-097	73.62	77.64	69.84	93.82	89.61	14
SNMOT-098	71.79	76.25	67.66	91.89	85.61	19
SNMOT-099	73.68	77.58	70.00	92.18	88.34	7
SNMOT-100	64.07	74.82	54.91	90.82	75.91	38
SNMOT-101	65.78	69.41	62.40	85.94	82.27	41
SNMOT-102	73.07	75.44	70.84	91.42	87.81	30
SNMOT-103	66.19	72.28	60.65	89.30	83.77	50
SNMOT-104	64.18	70.51	58.47	90.11	83.33	37
SNMOT-105	71.93	73.98	69.99	88.02	84.85	36
SNMOT-106	49.94	68.48	36.46	86.80	60.96	86
SNMOT-107	63.53	69.60	58.15	86.71	80.86	45
SNMOT-108	61.66	68.13	55.83	84.73	79.73	37
SNMOT-109	63.68	73.84	55.01	90.52	75.42	58
SNMOT-110	53.13	66.16	42.69	85.93	68.21	79
SNMOT-111	73.22	75.98	70.59	92.83	90.09	54
SNMOT-112	71.76	75.70	68.08	89.99	84.03	10
SNMOT-113	76.39	79.09	73.82	95.03	90.49	8
SNMOT-114	63.61	65.40	61.88	87.11	87.86	24
SNMOT-115	68.76	71.65	66.03	90.00	87.88	24
SNMOT-151	64.59	76.28	54.73	89.76	74.20	28
SNMOT-152	66.29	76.68	57.39	93.51	79.22	59
SNMOT-153	61.76	66.83	57.15	82.04	78.35	48
SNMOT-154	45.59	65.07	32.03	79.40	54.97	121
SNMOT-155	66.53	72.36	61.25	88.71	81.40	19
SNMOT-156	62.00	74.13	51.88	92.28	75.71	27
SNMOT-157	61.00	69.61	53.50	84.05	72.46	52
SNMOT-158	56.21	70.47	44.93	82.89	66.09	52
SNMOT-159	54.73	67.28	44.59	82.40	66.75	57
SNMOT-160	64.45	72.45	57.39	89.78	81.36	18
SNMOT-161	67.43	71.52	63.60	88.32	83.24	33
SNMOT-162	59.01	70.11	49.76	85.31	70.30	74
SNMOT-163	55.57	66.37	46.57	81.36	66.77	32
SNMOT-164	68.29	71.81	64.97	88.40	87.03	23
SNMOT-165	69.72	71.78	67.78	87.12	86.18	18
SNMOT-166	69.54	66.52	72.77	76.62	83.35	23
SNMOT-167	56.60	69.57	46.09	86.84	68.58	60
SNMOT-168	69.78	73.02	66.70	91.88	89.38	13
SNMOT-169	59.80	68.89	51.96	83.52	74.71	42
SNMOT-170	55.26	69.50	44.00	84.79	67.60	43
**COMBINED**	64.87	71.52	58.93	88.04	79.60	2268

**Table A2:** Per-sequence results of the final configuration (J) on the test set (49 sequences).

Sequence	HOTA	DetA	AssA	MOTA	IDF1	IDs
SNMOT-116	52.15	67.02	40.79	83.78	63.67	59
SNMOT-117	69.66	71.90	67.63	84.51	83.97	15
SNMOT-118	66.15	70.64	62.03	85.37	82.72	14
SNMOT-119	73.00	73.10	72.97	85.87	88.35	13
SNMOT-120	62.84	75.07	52.65	88.16	74.24	23
SNMOT-121	68.20	73.18	63.62	89.41	84.52	26
SNMOT-122	59.49	70.92	50.05	86.55	71.99	75
SNMOT-123	64.58	70.18	59.47	89.45	82.31	31
SNMOT-124	60.22	68.17	53.31	78.84	73.15	35
SNMOT-125	57.17	69.33	47.32	83.66	65.82	70
SNMOT-126	75.37	73.60	77.24	86.60	92.21	3
SNMOT-127	81.09	82.80	79.47	95.20	91.88	6
SNMOT-128	74.14	73.26	75.05	89.00	92.60	20
SNMOT-129	54.56	69.21	43.04	89.89	70.33	52
SNMOT-130	69.99	80.41	60.96	93.71	77.80	23
SNMOT-131	64.33	75.01	55.21	91.95	79.06	42
SNMOT-132	68.12	71.73	64.77	87.41	84.81	28
SNMOT-133	65.80	71.10	60.99	83.82	79.29	22
SNMOT-134	69.54	72.79	66.48	85.95	85.58	20
SNMOT-135	46.85	64.99	33.86	75.93	56.80	87
SNMOT-136	57.93	67.00	50.15	81.98	71.78	42
SNMOT-137	70.07	72.04	68.22	85.60	85.18	9
SNMOT-138	63.13	68.45	58.26	84.27	76.46	37
SNMOT-139	65.13	73.00	58.15	87.57	80.22	44
SNMOT-140	49.93	71.08	35.25	85.89	58.28	62
SNMOT-141	67.33	70.60	64.26	83.74	83.53	25
SNMOT-142	46.08	60.83	35.07	72.30	55.20	194
SNMOT-143	61.44	67.55	56.05	80.85	71.91	24
SNMOT-144	66.35	71.62	61.50	85.37	80.14	25
SNMOT-145	54.95	64.56	46.97	75.08	67.64	65
SNMOT-146	68.88	73.40	64.68	89.12	84.51	17
SNMOT-147	62.56	64.46	60.94	73.53	75.13	21
SNMOT-148	67.41	72.10	63.07	86.84	83.69	47
SNMOT-149	74.17	76.41	72.04	91.40	88.83	18
SNMOT-150	59.15	63.87	54.80	70.26	71.35	21
SNMOT-187	65.98	75.48	57.69	92.50	77.59	28
SNMOT-188	66.18	66.57	66.03	88.50	91.38	17
SNMOT-189	61.57	71.47	53.07	87.75	75.11	22
SNMOT-190	61.85	68.69	55.72	85.72	75.70	42
SNMOT-191	71.62	72.44	70.82	88.34	89.55	18
SNMOT-192	64.94	71.00	59.50	86.80	77.61	36
SNMOT-193	52.43	67.42	40.89	87.16	64.56	58
SNMOT-194	73.43	71.89	75.05	89.40	92.85	8
SNMOT-195	64.78	71.02	59.10	88.26	79.26	24
SNMOT-196	60.81	61.94	59.73	73.47	79.93	32
SNMOT-197	72.34	70.27	74.48	85.26	91.12	6
SNMOT-198	60.02	66.17	54.46	81.04	75.97	44
SNMOT-199	47.06	57.95	38.30	69.45	57.95	86
SNMOT-200	68.08	71.96	64.44	85.73	78.53	34
**COMBINED**	64.31	70.13	59.08	85.18	78.19	1770

**Table A3:** Statistical summary of the per-sequence results of the final configuration (J).

Split	Metric	Min	Max	Median	Mean	Std
Train (57)	HOTA	45.59	76.39	65.03	64.40	7.42
Train (57)	DetA	65.07	79.09	71.52	71.57	3.51
Train (57)	AssA	32.03	77.39	59.18	58.45	11.22
Train (57)	MOTA	76.62	95.03	88.32	87.95	3.88
Train (57)	IDF1	54.97	94.94	81.36	79.51	9.55
Train (57)	IDS	7.00	121.00	33.00	39.79	27.04
Test (49)	HOTA	46.08	81.09	64.94	63.85	7.80
Test (49)	DetA	57.95	82.80	71.02	70.32	4.53
Test (49)	AssA	33.86	79.47	59.50	58.48	11.35
Test (49)	MOTA	69.45	95.20	85.89	84.86	5.91
Test (49)	IDF1	55.20	92.85	79.06	78.00	9.81
Test (49)	IDS	3.00	194.00	26.00	36.12	30.82

**Table A4:** Pairwise statistical tests for the key HOTA differences reported in Section 5.3.1, Section 5.3.2, Section 5.3.3, Section 5.3.4 and Section 5.3.5 (test split unless the train split is noted). *W*: Shapiro–Wilk statistic on the per-sequence HOTA differences (α=0.05; normality not rejected for W>0.948 at n=49 and W>0.949 at n=57); paired-*t* when normality holds, otherwise Wilcoxon signed-rank. Cohen’s $d=\bar{\Delta }/\sigma_{\Delta }$; 95% CI: percentile bootstrap over $10^{4}$ resamples; $p_{\mathrm{adj}}$: Benjamini–Hochberg FDR at q=0.05. The per-sequence vectors of the final configuration are listed in Table A1 and Table A2; those of the remaining configurations are released with the code. The MCVC layer rows form a separate family and are corrected over the layer-ablation comparisons of the same split (15 on train, 14 on test; the identical +V3b edge is excluded); their $p_{\mathrm{adj}}$ is not comparable to that of the config-level rows.

Comparison	Split	*W*	Test (Stat)	$\bar{\Delta }$	Cohen’s *d*	95% CI	$p_{\mathrm{adj}}$
max_age 20 → 25	Test	0.973	t(48)=5.57	+2.80	+0.80	[+1.83, +3.77]	$2.5\times 10^{-6}$
max_age 25 → 30	Test	0.981	t(48)=−6.16	−3.12	−0.88	[−4.11, −2.11]	$5.3\times 10^{-7}$
max_age 30 → 60	Test	0.874	Wilcoxon W=479	−0.14	−0.15	[−0.42, +0.12]	0.290
max_age 60 → 100	Test	0.813	Wilcoxon W=510	+0.05	+0.05	[−0.21, +0.31]	0.739
max_age 20 → 25	Train	0.645	Wilcoxon W=180	−0.12	−0.41	[−0.20, −0.05]	0.006
max_gap 250 → 350	Train	0.952	Wilcoxon W=472	−0.12	—	[−0.20, +0.20]	0.253
max_age 25 → 30	Train	0.667	Wilcoxon W=334	+0.03	+0.06	[−0.11, +0.17]	0.792
conf 0.25 → 0.30	Test	0.967	t(48)=−5.47	−3.28	−0.78	[−4.46, −2.12]	$2.9\times 10^{-6}$
conf 0.25 → 0.30	Train	0.923	Wilcoxon W=566	−0.58	−0.30	[−1.10, −0.11]	0.067
conf 0.30 → 0.35	Test	0.911	Wilcoxon W=194	−1.56	−0.68	[−2.20, −0.94]	$1.6\times 10^{-5}$
conf 0.30 → 0.35	Train	0.979	t(56)=−3.17	−0.96	−0.42	[−1.55, −0.36]	0.006
imgsz 960 → 1280	Test	0.976	t(48)=4.32	+1.93	+0.62	[+1.06, +2.79]	$9.4\times 10^{-5}$
imgsz 1280 → 1536	Test	0.939	Wilcoxon W=101	−3.83	−0.88	[−5.09, −2.65]	$1.1\times 10^{-7}$
n_init 2 → 3	Test	0.974	t(48)=7.65	+3.71	+1.09	[+2.79, +4.65]	$8.2\times 10^{-9}$
n_init 2 → 3	Train	0.901	Wilcoxon W=208	+1.19	+0.70	[+0.76, +1.65]	$6.2\times 10^{-6}$
match_thresh 0.60 → 0.70	Test	0.978	t(48)=4.96	+2.82	+0.71	[+1.73, +3.94]	$1.5\times 10^{-5}$
match_thresh 0.60 → 0.70	Train	0.928	Wilcoxon W=648	−0.14	−0.09	[−0.56, +0.29]	0.182
match_thresh 0.70 → 0.80	Test	0.979	t(48)=−5.72	−3.05	−0.82	[−4.08, −2.03]	$1.8\times 10^{-6}$
match_thresh 0.70 → 0.80	Train	0.951	Wilcoxon W=580	−0.44	−0.28	[−0.86, −0.05]	0.070
Config F → G	Test	0.971	t(48)=4.83	+3.03	+0.95	[+1.78, +4.28]	<0.001
Config B → D	Test	—	≡ 0.00	0.00	0.00	[0.00, 0.00]	—
Config F → J (MCVC)	Test	0.968	t(48)=7.94	+3.43	+1.13	[+2.57, +4.29]	<0.001
StrongSORT re-run → I	Test	0.974	t(48)=9.71	+1.79	+1.38	[+1.64, +1.94]	<0.001
Config I → J	Test	0.921	Wilcoxon W=105	+1.34	+0.82	[+0.90, +1.80]	$2.7\times 10^{-8}$
Config I → J	Train	0.918	Wilcoxon W=202	+1.21	+0.75	[+0.80, +1.63]	$7.0\times 10^{-7}$
MCVC: nominal win. → veto	Train	0.910	Wilcoxon W=196	+1.23	+0.76	[+0.83, +1.66]	$7.6\times 10^{-6}$
MCVC: nominal win. → veto	Test	0.918	Wilcoxon W=110	+1.37	+0.85	[+0.94, +1.84]	$5.7\times 10^{-7}$
MCVC: cone pruning only	Train	0.879	Wilcoxon W=347	+0.11	+0.21	[−0.03, +0.25]	0.129
MCVC: veto → +cone pruning	Train	0.875	Wilcoxon W=410	+0.05	+0.08	[−0.11, +0.19]	0.350
MCVC: veto → +cone pruning	Test	0.690	Wilcoxon W=251	−0.07	−0.20	[−0.18, +0.02]	0.498
MCVC: +consensus (V3a)	Train	0.944	Wilcoxon W=700	−0.03	−0.05	[−0.21, +0.14]	0.959
MCVC: +purity (V3b)	Train	—	≡0.00	0.00	0.00	[0.00, 0.00]	—
MCVC: +recovery (T4-400)	Train	0.920	Wilcoxon W=542	−0.20	−0.26	[−0.40, −0.00]	0.129
MCVC: +all layers	Train	0.937	Wilcoxon W=487	−0.30	−0.33	[−0.54, −0.07]	0.052
MCVC: veto → +all layers	Test	0.945	Wilcoxon W=445	−0.16	−0.19	[−0.40, +0.09]	0.158
ByteTrack → Ours (J)	Test	0.973	t(48)=8.92	+10.41	+1.55	[+9.08, +11.74]	<0.001
OC-SORT → Ours (J)	Test	0.969	t(48)=9.62	+13.84	+1.62	[+11.97, +15.71]	<0.001
BoT-SORT → Ours (J)	Test	0.971	t(48)=8.05	+9.76	+1.45	[+8.36, +11.16]	<0.001
StrongSORT → Ours (J)	Test	0.965	t(48)=6.84	+2.51	+1.05	[+1.79, +3.23]	<0.001
Kalisteo (challenge) → Ours (test)	—	—	split-mismatch	—	—	—	n/a
